# Exosome Release Delays Senescence by Disposing of Obsolete Biomolecules

**DOI:** 10.1002/advs.202204826

**Published:** 2023-01-22

**Authors:** Wenchong Zou, Mingqiang Lai, Yuanjun Jiang, Linlin Mao, Wu Zhou, Sheng Zhang, Pinglin Lai, Bin Guo, Tiantian Wei, Chengtao Nie, Lei Zheng, Jiahuan Zhang, Xuefei Gao, Xiaoyang Zhao, Laixin Xia, Zhipeng Zou, Anling Liu, Shiming Liu, Zhong‐Kai Cui, Xiaochun Bai

**Affiliations:** ^1^ Guangdong Provincial Key Laboratory of Bone and Joint Degenerative Diseases The Third Affiliated Hospital of Southern Medical University Guangzhou 510630 China; ^2^ State Key Laboratory of Organ Failure Research Department of Cell Biology School of Basic Medical Sciences Southern Medical University Guangzhou 510515 China; ^3^ The Fifth Affiliated Hospital Southern Medical University Guangzhou Guangdong 510900 China; ^4^ Department of Laboratory Medicine Nanfang Hospital Southern Medical University Guangzhou 510515 China; ^5^ Department of Physiology School of Basic Medical Sciences Southern Medical University Guangzhou 510515 China; ^6^ Department of Developmental Biology School of Basic Medical Sciences Southern Medical University Guangzhou 510515 China; ^7^ Department of Biochemistry School of Basic Medical Sciences Southern Medical University Guangzhou 510515 China; ^8^ Department of Cardiology Guangzhou Institute of Cardiovascular Disease Guangdong Key Laboratory of Vascular Diseases The Second Affiliated Hospital Guangzhou Medical University Guangzhou 510260 China; ^9^ Department of Spine Surgery Ganzhou People's Hospital Ganzhou 342800 China

**Keywords:** aging, exosomes, mTORC1, nutrient restriction, obsolete biomolecules

## Abstract

Accumulation of obsolete biomolecules can accelerate cell senescence and organism aging. The two efficient intracellular systems, namely the ubiquitin‐proteasome system and the autophagy‐lysosome system, play important roles in dealing with cellular wastes. However, how multicellular organisms orchestrate the processing of obsolete molecules and delay aging remains unclear. Herein, it is shown that prevention of exosome release by GW4869 or *Rab27a* ^−/−^ accelerated senescence in various cells and mice, while stimulating exosome release by nutrient restriction delays aging. Interestingly, exosomes isolate from serum‐deprived cells or diet‐restricted human plasma, enriched with garbage biomolecules, including misfolded proteins, oxidized lipids, and proteins. These cellular wastes can be englobed by macrophages, eventually, for disintegration in vivo. Inhibition of nutrient‐sensing mTORC1 signaling increases exosome release and delays senescence, while constitutive activation of mTORC1 reduces exosome secretion and exacerbates senescence in vitro and in mice. Notably, inhibition of exosome release attenuates nutrient restriction‐ or rapamycin‐delayed senescence, supporting a key role for exosome secretion in this process. This study reveals a potential mechanism by which stimulated exosome release delays aging in multicellular organisms, by orchestrating the harmful biomolecules disposal via exosomes and macrophages.

## Graphical abstract

Exosome release from cells is closely correlated with a senescent phenotype both in vitro and in vivo. Inhibition of exosome release accelerates, while stimulation of exosome secretion delays cell senescence and organism aging. Increased exosome secretion by nutrient restriction or rapamycin allows the excretion of harmful biomolecules which can be disposed of by professional “cleaners”, such as macrophages.

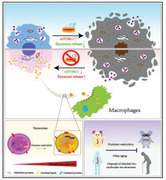

## 1 Introduction

How to delay aging has always been pursued by people, and scientists have thus been committed to unravelling the mechanisms underlying aging and investigating approaches to delaying senescence.^[^ ^1^ ^]^ The biological functions of cells and orgnisms generally rely on the correct folding of proteins, which in turn depends on the amino acid sequences.^[^ ^2^ ^]^ In normal cells, misfolded proteins are rapidly degraded to prevent toxicity due to the accumulation and aggregation of these protein fragments.^[^ ^3^ ^]^ Two main mechanisms of intracellular protein degradation are well‐acknowledged: the ubiquitin‐proteasome system and the autophagy‐lysosome system.^[^ ^4^ ^]^ However, both systems may not be efficient enough to degrade misfolded proteins prior to their aggregation in senescent cells^[^ ^2^, ^5^ ^]^ Preventing their degradation then accelerates senescence and shortens lifespan, leading to aging and age‐related diseases.^[^ ^6^ ^]^ Multiple age‐related diseases, including neurodegeneration, diabetes, cystic fibrosis, and cardiac amyloidosis, mainly or partially result from the inability of cells to degrade misfolded or damaged proteins.^[^ ^7^ ^]^ The two classic degradation systems mostly function within cells, while multicellular organisms possess various types of cells, and how such organisms orchestrate the processing of obsolete molecules, and therefore delay aging is largely unknown.

Despite the complex and multifactorial antiaging mechanisms, some key nodes in a highly conserved regulatory network have been discovered.^[^ ^8^ ^]^ Mechanistic target of rapamycin complex 1 (mTORC1) has been shown to act as a robust nutrient‐sensing node for aging signals in laboratory models and in some preliminary clinical studies.^[^ ^9^ ^]^ Inhibition of mTORC1 delayed aging in yeast, Drosophila, *Caenorhabditis elegans*, and mice, simulating the effects of nutrient restriction on senescence.^[^ ^10^ ^]^ Previous mechanistic studies indicated that inhibition of mTORC1 limited protein synthesis and endoplasmic reticulum (ER) stress, reduced the production of reactive oxygen species (ROS), stem cell proliferation, and exhaustion, and notably promoted autophagy to maintain cell health and delay cell senescence.^[^ ^11^ ^]^ However, how mTORC1 activation promotes, while its inhibition delays aging in multicellular organisms is less understood.

Exosomes are membrane‐bound extracellular vesicles that are produced in the endosomal compartment of most eukaryotic cells under physiological and pathological conditions.^[^ ^12^ ^]^ They contain signaling proteins, miRNAs, mRNAs, DNA, and lipids that are capable of regulating the cellular activities of recipient cells.^[^ ^13^ ^]^ Therefore, exosomes are well‐known key players in cell communication, among neighbors and in distant tissues.^[^ ^14^ ^]^ However, the correlation of exosome release and senescence in cells and multicellular organisms remains unexplored.

In this study, we demonstrated that inhibition of exosome release accelerated senescence both in vitro and in vivo, while inhibition of mTORC1 promotes the secretion of exosomes containing obsolete molecules, resulting in delayed aging. We found that increased exosome secretion allowed the excretion of harmful biomolecules that cannot be processed within cells, but which can be disposed of by professional “cleaners”, such as macrophages. Our findings identify exosome release as an alternative and essential waste‐disposing approach, representing a novel perspective underlying the antiaging mechanism in multicellular organisms.

## 2 Results

### 2.1 Inhibition of Exosome Release Accelerates Senescence In Vitro and In Mice

**Figure 1:**
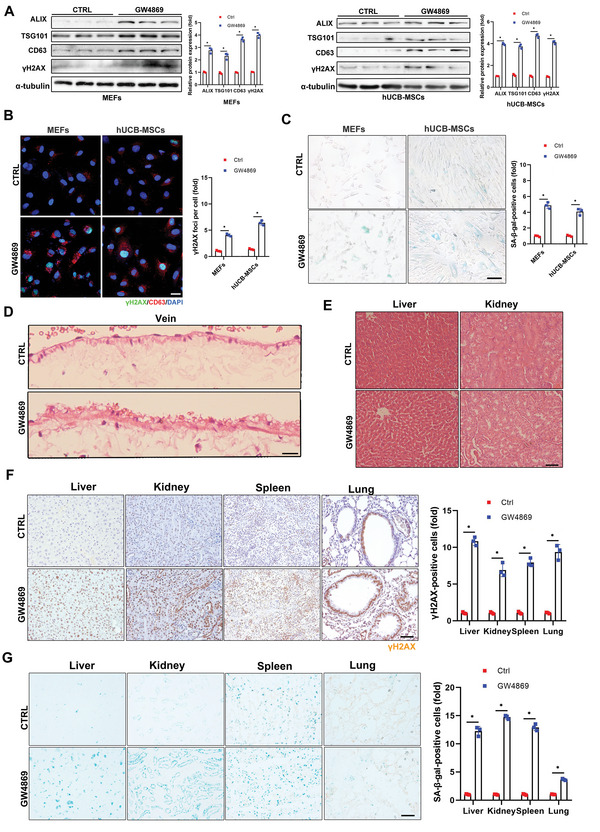
Inhibition of exosome release accelerates senescence in vitro and in mice. A–D) Mouse embryonic fibroblasts (MEFs) and human umbilical cord blood mesenchymal stem cells (hUCB‐MSCs) were treated with GW4869 (2 × 10^−6^ m) for 5 d. A) Cell levels of exosome marker proteins and senescence marker proteins were determined by western blot, with quantification. Three independent experiments were analyzed. B) Cells were fixed and stained with anti‐*γ*H2AX (green) and anti‐CD63 antibodies (red), and nuclei were stained with DAPI (blue). Confocal microscopic images of stained cells, with quantitative presentation of fold‐changes in *γ*H2AX‐positive foci in cells. Scale bars = 30 µm. A total of 60 randomly selected cells from three independent experiments were analyzed. C) Cells were fixed and stained with SA‐*β*‐gal. Scale bars = 50 µm. SA‐*β*‐gal‐positive cells were quantified. A total of 60 randomly selected cells from three independent experiments were analyzed. D–G) Six‐month‐old mice (*n* = 3) were injected with GW4869 (2.5 µg g^−1^) or drug vehicle for 2 months via the tail vein. The animals were sacrificed and tissues were collected. D,E) Representative hematoxylin and eosin‐stained images of veins, liver, and kidney. Vein, scale bars = 30 µm; liver and kidney, scale bars = 50 µm. F) Tissues were fixed and stained with anti‐*γ*H2AX antibody and G) SA‐*β*‐gal. Scale bars = 50 µm. Relative positive cells were quantified. *p < 0.05.

To explore the effects of inhibiting exosome release on cell senescence, three types of primary cultured cells, namely mouse embryonic fibroblasts (MEFs), human umbilical cord blood mesenchymal stem cells (hUCB‐MSCs), and human umbilical vein endothelial cells (HUVECs) were used. The inhibitor of exosome release, GW4869, was used to suppress exosome release both in vitro and in mice.^[^ ^15^ ^]^ The presence of exosomes within cells was verified by increased protein expression of the biomarkers CD63, ALG‐2‐interacting protein X (ALIX), and tumor susceptibility gene 101 (TSG101), and significantly increased expression levels of *γ*H2AX, as an early cellular response to the induction of DNA double‐strand breaks^[^ ^16^ ^]^ (**Figure** **1**A,B; Figure S1, Supporting Information). Senescence‐associated *β*‐galactosidase (SA‐*β*‐gal) expression was intensified in all three cell types following the addition of GW4869 (Figure 1C; Figure S1C, Supporting Information), and mRNA expression levels of *IL6*, *MMP13*, and *P16* were increased while that of *lamin B1* was decreased (Figure S1D, Supporting Information). Overall, inhibition of exosome release from cells in vitro led to senescent phenotypes.

Similar effects were observed in mice injected with GW4869 via the tail vein for 2 months, to suppress exosome release (Figure S1E,F, Supporting Information). This resulted in accelerated aging, based on increased thickness of the veins (Figure 1D) and vacuolation of cells in the liver and kidney (Figure 1E), but no significant difference in the spleen or lung (Figure S1G, Supporting Information). Intensified staining of *γ*H2AX and SA‐*β*‐gal in liver, kidney, spleen, and lung sections further confirmed the senescent features in mice (Figure 1F,G) following inhibition of exosome secretion. P16 and lamin B1 immunostaining were also consistent with the senescent characteristics (Figure S1H,I, Supporting Information).

**Figure 2:**
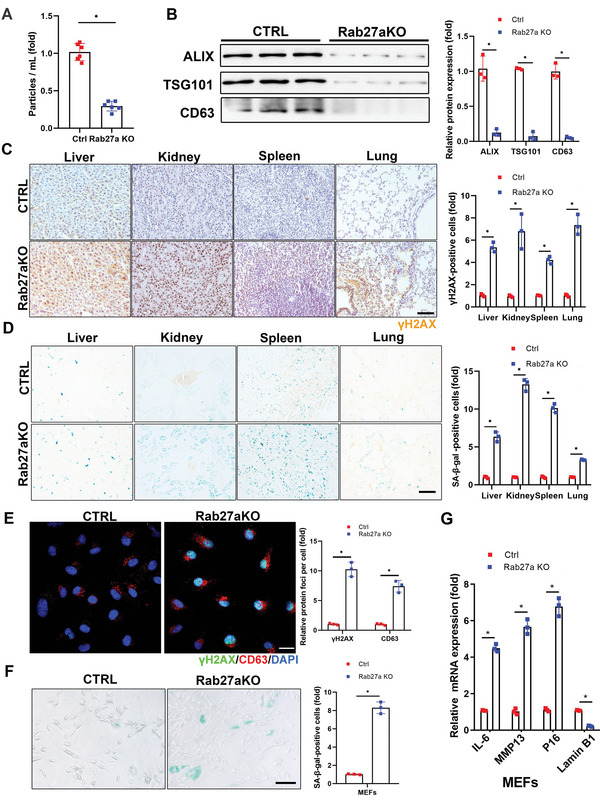
Deletion of *Rab27a* prevents exosome release and accelerates senescence in cultured cells and in mice. A) *Rab27a* KO mice (6‐months‐old, *n* = 6) were sacrificed and plasma exosomes were isolated and subjected to NTA. B) Levels of exosome marker proteins in released exosomes isolated from mouse plasma were determined by western blot, with quantification. Three independent experiments were analyzed. C) Tissues were fixed and stained with anti‐*γ*H2AX antibody and D) SA‐*β*‐gal. Scale bars = 50 µm. Relative positive cells were quantified. Three independent experiments were analyzed. E) Confocal microscopic images of stained cells. *Rab27a* KO MEFs were fixed and stained with anti‐*γ*H2AX (green) and anti‐CD63 antibodies (red), and nuclei were stained with DAPI (blue), with quantitative presentation of the fold‐changes of *γ*H2AX‐positive foci in cells. Scale bars = 30 µm. A total of 60 randomly selected cells from three independent experiments were analyzed. F) *Rab27a* KO MEFs were fixed and stained with SA‐*β*‐gal. Scale bars = 50 µm. SA‐*β*‐gal‐positive cells were quantified. A total of 60 randomly selected cells from three independent experiments were analyzed. G) Expression of *IL6, P16, MMP13*, and *Lamin B1* mRNA in *Rab27a* KO MEFs were determined by qRT‐PCR. *GAPDH* was used as the internal control. Three independent experiments were analyzed. *p < 0.05.

Rab27a has been shown to play an important role in exosome secretion.^[^ ^17^, ^18^ ^]^ We systematically knocked out the *Rab27a* gene in mice to construct an inherent exosome‐release‐inhibition in vivo model (Figure S2A,B, Supporting Information). The total number of exosomes extracted via ultracentrifugation from the plasma of adult (6‐month‐old) *Rab27a*‐knockout (KO, *Rab27a* ^−/−^) mice was remarkably decreased compared with control mice of the same age (**Figure** **2**A,B). *γ*H2AX and SA‐*β*‐gal expression levels in the major organs were increased in *Rab27a^−/−^* mice (Figure 2C,D). Although there were no notable alterations in the microstructures of the harvested organs (Figure S2C, Supporting Information), intensified P16 and decreased lamin B1 staining indicated senescent features in these major organs in *Rab27a^−/−^* mice (Figure S2D,E, Supporting Information).

We further clarified the effect of *Rab27a* deletion on cell senescence using primary MEFs extracted from *Rab27a^−/−^* mouse embryos. As expected, exosomes confined within MEFs were significantly increased, as indicated by CD63 staining (Figure 2E). *γ*H2AX and SA‐*β*‐gal staining were markedly intensified (Figure 2E,F; passage 6) and *IL6, MMP13*, and *P16* mRNA expression levels were increased, while that of *lamin B1* was decreased in *Rab27a^−/−^* compared with control MEFs (Figure 2G). However, younger MEFs (passage 2) showed no obvious senescent phenotype or variations in senescent biomarkers (Figure S2F–H, Supporting Information). Collectively, the exosome‐secretion inhibitor, GW4869, and deletion of *Rab27a* both suppressed exosome release in cultured cells and in mice, leading to senescent characteristics and accelerated aging.

### 2.2 Exosome Release Delays Aging via Expelling Obsolete Biomolecules

**Figure 3:**
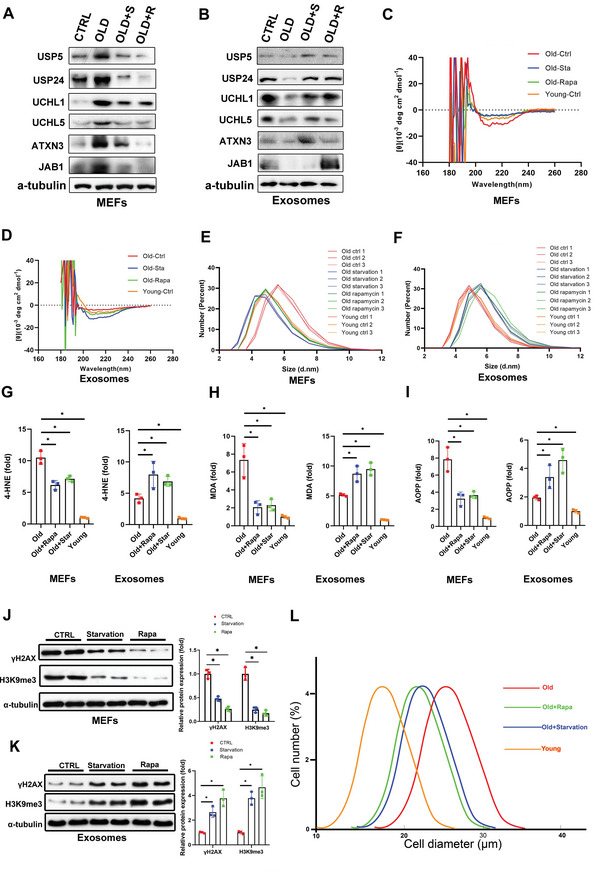
Nutrient restriction and rapamycin delay aging by stimulating exosome release to expel obsolete biomolecules. A,B) Levels of deubiquitinases in MEFs and secreted exosomes were determined by western blot. Young (passage 2) and old (passage 8) MEFs were treated with rapamycin (100 × 10^−9^ m) or cultured in serum‐deprived medium for 24 h. C) Cell lysates and D) exosomes isolated from the culture media were subjected to circular dichroism analysis (*n* = 3). E) Cell lysates and F) exosomes isolated from the culture media were subjected to dynamic light scattering analysis (*n* = 3). Cell lysates and exosomes were subjected to evaluation of G) 4‐HNE, H) MDA, and I) AOPP. Three independent experiments were analyzed. *p < 0.05. J) Cell lysates and K) exosomes isolated from the culture media were assessed by western blot, with quantification. Three independent experiments were analyzed. L) The size distribution within the population of cells was measured using an MS3 Coulter counter. *p < 0.05 (average of n = 3 replicates, with 13 750 cells each).

Ubiquitination generally tags proteins for recognition by the proteasome for degradation, and aging leads to a global loss of ubiquitination because of increased deubiquitinase activity.^[^ ^19^ ^]^ Therefore, deubiquitinases in cells and exosomes were examined and the results showed that deubiquitinases, including USP5, USP24 (the ubiquitin‐specific protease (USP/UBP) superfamily), UCHL1, UCHL5 (the ubiquitin C‐terminal hydrolase (UCH) superfamily), ATXN3 (the Machado–Josephin domain superfamily), and JAB1 (the JAMM domain superfamily), in old MEFs (passage 8) were markedly increased, while decreased in the corresponding cell‐secreted exosomes (**Figure** **3**A,B). After serum starvation (1% fetal bovine serum, FBS) or rapamycin treatment, the amount of deubiquitinases in old MEFs significantly reduced, while augmented in the corresponding cell‐secreted exosomes (Figure 3A,B). We therefore propose that misfolded and damaged proteins might be bagged by the cells for disposal, to maintain homeostasis when the workload exceeds the processing capability of the ubiquitin‐proteasome system.

To further test our hypothesis, we monitored the conformational changes of proteins inside the cells and cell‐secreted exosomes using circular dichroism spectroscopy, to reveal their secondary structural characteristics. Misfolded proteins typically contain *β*‐sheets organized in a supramolecular arrangement known as a cross‐*β* structure.^[^ ^20^ ^]^ These *β*‐sheet‐rich assemblies are very stable, highly insoluble, and generally resistant to proteolysis.^[^ ^21^ ^]^ Misfolding of proteins can trigger further misfolding and the accumulation of other proteins into aggregates or oligomers.^[^ ^22^ ^]^ Compared with normal young cells (passage 2), senescent cells (passage 8) comprised more proteins containing *β*‐sheets at around 216 and 195 nm, respectively (Figure 3C). Serum starvation or rapamycin treatment for 24 h significantly reduced the amount of proteins containing *β*‐sheets in senescent cells compared with normal levels (Figure 3C). Concomitantly, the amount of proteins containing *β*‐sheets in cell‐secreted exosomes tended to be opposite but complementary to the results for intracellular protein conformation (Figure 3D).

The granularity of the particles (mainly proteins) within cells and cell‐secreted exosomes was evaluated using a Malvern nanosizer. Granularity was significantly higher in older cells (passage 8) compared with younger cells (passage 2) (Figure 3E). However, serum deprivation and rapamycin treatment of old cells decreased their granularity, similar to the level in young cells (Figure 3E). Complementary results were observed in the corresponding exosomes secreted from old cells (Figure 3F). Particle granularity was increased in exosomes isolated from old cells treated with serum deprivation or rapamycin, inferring that aggregated particles were bagged by exosomes for disposal.

The production of ROS and free radicals is increased during aging, leading to oxidative damage.^[^ ^23^ ^]^ Lipid peroxidation generates reactive aldehydic products, such as 4‐hydroxynonenal (HNE) and malondialdehyde (MDA), that modify proteins and form adducts with DNA bases.^[^ ^24^ ^]^ Advanced oxidation protein products (AOPP) are a sensitive marker of oxidative stress.^[^ ^25^ ^]^ Interestingly, 4‐HNE, MDA, and AOPP levels were significantly decreased in senescent cells subjected to starvation or rapamycin treatment, and increased in secreted exosomes (Figure 3G–I). Cellular senescence is also considered to be a consequence of the accumulation of genetic errors, including DNA misduplication, gene mistranscription and mistranslation, and DNA misrepair.^[^ ^26^ ^]^ Accordingly, the nuclear proteins *γ*H2AX, a sensitive marker of DNA damage and H3K9me3^[^ ^27^ ^]^ levels were significantly decreased in senescent cells and increased in exosomes isolated from the corresponding cells after serum starvation and rapamycin treatment (Figure 3J,K). These findings indicate that nutrient restriction and rapamycin accelerate exosome release to expel damaged DNA, obsolete lipid, and protein peroxidative products.

Senescent cells are usually larger than non‐senescent ones.^[^ ^28^ ^]^ We therefore measured the diameters of young and old cells (Figure 3L). The average diameter of old cells was 26 µm compared with 18 µm of young cells. However, old cells with serum deprivation or rapamycin treatment (increase of exosomes release) reduced their diameter to around 21 µm, indicating that enhanced exosome release could partly reduce cell size to reverse the senescent phenotype.

Overall, these results indicate that the release of exosomes stimulated by nutrient restriction can expel obsolete biomolecules, including misfolded proteins, damaged lipids, and possibly harmful DNA, to maintain cell homeostasis.

### 2.3 Exosomes Carry the “Garbage” to Macrophages for Disposal and Disintegration In Vivo

**Figure 4:**
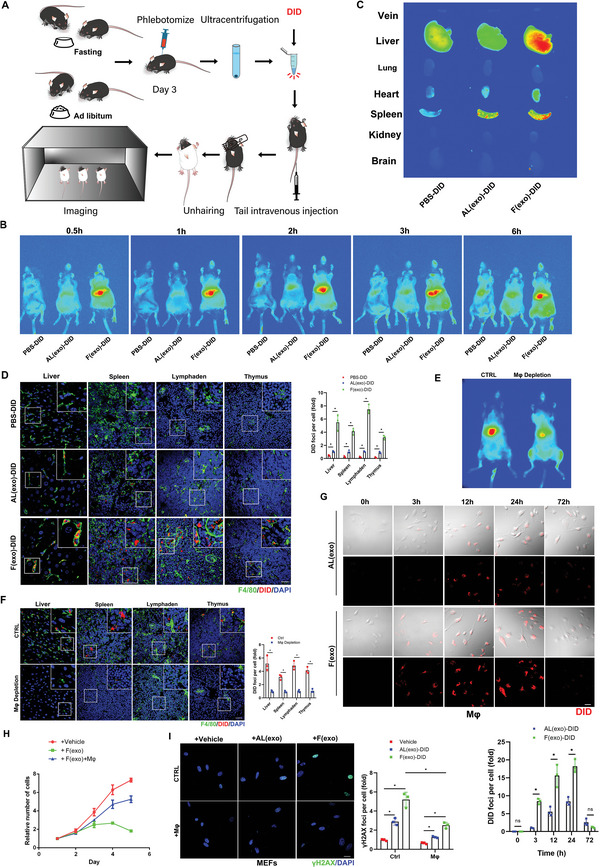
Exosomes carry “garbage” to macrophages for disposal and disintegration. A) Schematic illustration of exosomes isolated from mouse plasma (with/without 2‐d fasting) labeled with DiD (hydrophobic fluorescent dye) for tracking. B) The same numbers of exosomes isolated from fasting or control groups, labeled with DiD dye (red) were injected into mice via the tail vein for in vivo imaging analysis. C) The mice were sacrificed and vein, liver, lung, heart, spleen, kidney, and brain tissues were collected and imaged. D) Confocal microscopic images of stained tissues. Liver, spleen, thymus, and lymph nodes were fixed with paraformaldehyde and frozen sections were prepared and stained with anti‐F4/80 (green), and nuclei were stained with DAPI (blue), with quantitative presentation of the fold‐changes of DiD‐positive foci in cells. Scale bars = 30 µm. A total of 60 randomly selected cells from three independent experiments were analyzed. E) Mice were injected with a macrophage scavenger and exosomes isolated from the plasma of mice fasted for 2 d (6‐months‐old) and labeled with DiD were injected into mice via the tail vein for in vivo imaging. F) The mice (n = 3) were sacrificed and liver, spleen, thymus, and lymph node tissues were collected and fixed with paraformaldehyde, and frozen sections were prepared and stained with anti‐F4/80 (green), and nuclei were stained with DAPI (blue), with quantitative presentation of the fold‐changes of DiD‐positive foci in cells. Scale bars = 30 µm. *p < 0.05. (G) Confocal microscopic images of stained cells. Primary macrophages were extracted from mouse bone marrow. Exosomes isolated from mouse plasma (with/without 2‐d fasting) labeled with DID (red) were cocultured with primary macrophages for 72 h, with quantitative presentation of fold‐changes of DiD‐positive foci in cells. Scale bars = 30 µm. A total of 60 randomly selected cells from three independent experiments were analyzed. H) Primary MEFs (passage 4) were cocultured with exosomes extracted from mice (with 2‐d fasting) and macrophages for 5 d. The proliferation ability of cells in each group was determined by CCK8 assay. Three independent experiments were analyzed. I) The cells were fixed and stained with anti‐*γ*H2AX (green) and DAPI staining for nuclei (blue), with quantitative presentation of the fold‐change of *γ*H2AX‐positive foci in cells. A total of 60 randomly selected cells from three independent experiments were analyzed. Scale bars = 30 µm. *p < 0.05.

To determine the fate of the secreted exosomes and the identity of the “cleaners” responsible for degrading the “garbage” biomolecules in multicellular organisms, exosomes isolated from plasma of 6‐month‐old mice with/without 2‐d fasting were labeled with DiD (lipophilic fluorescent dye) for tracking (**Figure** **4**A). The same number of exosomes (4×10^10^ particles mL^−1^, 100 µL) isolated from the Fasting or Ctrl group (food ad libitum) were injected into 2‐month‐old mice via the tail vein. Exosomes were mainly located in the liver and spleen, with enhanced accumulation in the Fasting group compared with the Ctrl group over the investigated time range (0–6 h) (Figure 4B,C). The higher uptake of exosomes, isolated from the fasted group compared to the control group, by macrophages in the recipient mice probably resulted from the high expressions of phosphatidylserine^[^ ^29^ ^]^ and fibronectin^[^ ^30^ ^]^ on the membrane (Figure S3, Supporting Information). Liver, spleen, lymph nodes, and thymus were harvested, frozen‐sectioned, and stained with the macrophage marker F4/80. Confocal microscopic images revealed that exosomes were phagocytosed by macrophages in all the above organs (Figure 4D). When the macrophages were eliminated in the liver, the exosomes in the Fasting group tended to distribute throughout the body (Figure 4E) and were no longer phagocytosed by macrophages in the liver, spleen, lymph nodes, or thymus (Figure 4F). Macrophages were thus revealed as one of the key destinations for nutrient restriction‐stimulated exosomes.

We showed that obsolete biomolecules were expelled via exosomes to delay senescence, and those exosomes circulated to macrophages. We therefore investigated the role of macrophages as possible “cleaners” to process the “garbage”. We cocultured macrophages with DiD‐labeled exosomes for 72 h and showed that macrophages took up the exosomes by endocytosis, with maximum red fluorescence at 24 h. Exosomes from the Fasting group were almost digested by the macrophages by 72 h (Figure 4G). Primary MEFs (passage 4) cocultured with exosomes extracted from Fasting mice and macrophages for 5 d showed relatively high proliferation, while MEFs cocultured with exosomes without macrophages were more prone to senescence and showed significantly decreased proliferation (Figure 4H). *γ*H2AX staining exhibited consistent results (Figure 4I).

**Figure 5:**
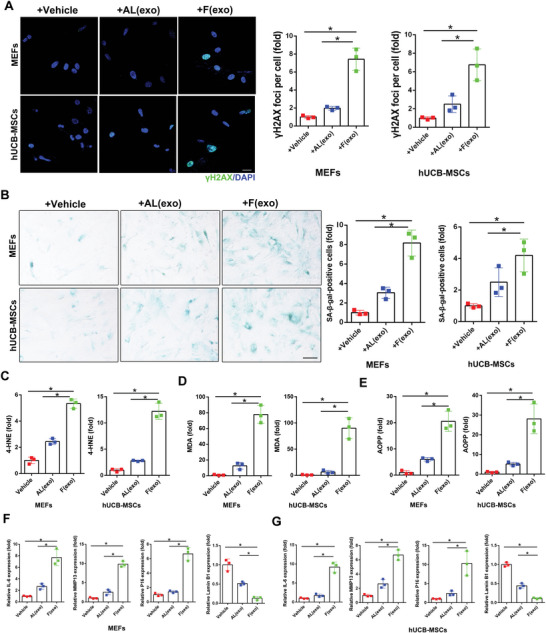
Exosomes isolated from mouse and human plasma after fasting accelerate cell senescence. MEFs (Passage 2) and hUCB‐MSCs uptook exosomes isolated frommouse and human plasma before (+AL) and after (+F)_fasting, respectively. A) Confocal microscopic images of stained cells. Cells were fixed and stained with anti‐*γ*H2AX (green) and nuclei were stained with DAPI (blue), with quantitative presentation of the fold‐changes of *γ*H2AX‐positive foci in cells. Scale bars = 30 µm. A total of 60 randomly selected cells from three independent experiments were analyzed. B) Cells were fixed and stained with SA‐*β*‐gal. Scale bars = 50 µm. SA‐*β*‐gal‐positive cells were quantified. A total of 60 randomly selected cells from three independent experiments were analyzed. Cell lysates and exosomes were subjected to evaluation of C) 4‐HNE, D) MDA, and E) AOPP. Three independent experiments were analyzed. F,G) Expression of *IL6, P16, MMP13*, and *Lamin B1* mRNA in cells were determined by qRT‐PCR. *GAPDH* was used as the internal control. Three independent experiments were analyzed. *p < 0.05.

Similar results were also observed in exosomes isolated from mouse and human plasma after fasting, which accelerated MEF and hUCB‐MSC senescence (**Figure** **5**), evidenced by increased *γ*H2AX and SA‐*β*‐gal staining (Figure 5A,B), significantly augmented oxidative adducts (Figure 5C–E) and correspondingly altered senescent‐associated mRNA levels (Figure 5F,G). These findings indicate that nutrient restriction stimulated exosome release to expel obsolete biomolecules that could accelerate senescence, at least in vitro, while macrophages acted as “cleaners” to disintegrate the “garbage” delivered by the exosomes.

### 2.4 Activation of mTORC1 Prevents Exosome Release and Promotes Senescence

mTORC1 functions as a nutrient/energy sensor.^[^ ^31^ ^]^We therefore examined the role of mTORC1 in nutrient‐regulated exosome secretion and senescence in mice. The mice were divided randomly into Ctrl, Fasting, and Recovery groups. No food intake for 2 d in the Fasting group resulted in significantly decreased phosphorylation of S6 ribosomal protein (pS6)^[^ ^32^ ^]^ in the major organs (Figure S5A,B, Supporting Information), indicating reduced mTORC1 activity following nutrient restriction. pS6 levels increased in the Recovery group (24 h after normal food intake following 2 d of fasting), comparable to the Ctrl group. Similar trends in mTORC1 activity were observed in cultured cells (MEFs, hUCB‐MSCs, HUVECs), with lower pS6 expression due to serum deprivation in the Starvation group, while serum replenishment increased pS6 levels in the +Serum group (Figure S5C, Supporting Information). These results confirmed that nutrient restriction and serum deprivation inhibited mTORC1 activity in mice and cultured cells, respectively.

**Figure 6:**
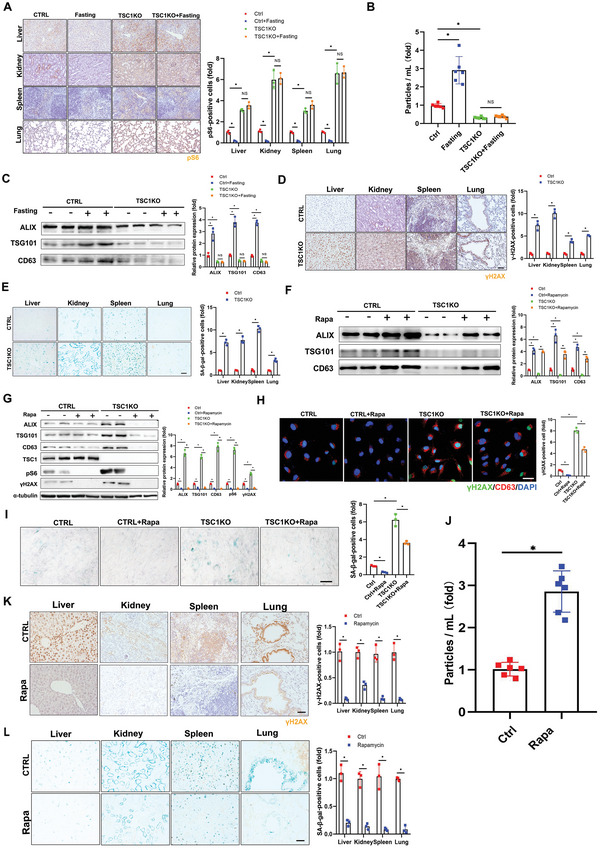
Nutrient‐sensing mTORC1 prevents exosome release and promotes senescence. A–C) Three‐month‐old tamoxifen‐inducible UBC‐Cre‐ERT2; *TSC1* fl/fl mice (*TSC1* KO, *n* = 6) were treated with tamoxifen (100 mg/kg/day) for 5 d before fasting for 2 d. A) The animals were sacrificed and tissues were collected, fixed, and stained with anti‐pS6 antibody for immunohistochemical analysis. Scale bars = 50 µm. Three independent experiments were analyzed. B) Plasma exosomes were isolated and subjected to NTA. Six independent experiments were analyzed. C) Levels of exosome marker proteins in plasma exosomes were determined by western blot, with quantification. Three independent experiments were analyzed. D,E) Three‐month‐old tamoxifen‐inducible UBC‐Cre‐ERT2; *TSC1* fl/fl mice (*TSC1* KO, *n* = 3) were sacrificed and tissues were collected, fixed, and stained with (D) anti‐*γ*H2AX antibody and (E) SA‐*β*‐gal. Scale bars = 50 µm. Three independent experiments were analyzed. Relative positive cells were quantified. F–H) Tamoxifen‐inducible UBC‐Cre‐ERT2; *TSC1* fl/fl primary MEFs were treated with 4‐OHT (10 × 10^−6^ m) for 3 d followed by rapamycin (100 × 10^−9^ m) or vehicle control for 24 h. F) Levels of exosome marker proteins in released exosomes and G) cell extracts were determined by western blot. mTORC1 activity in the cells was monitored with levels of pS6 by western blot, with quantification. Three independent experiments were analyzed. H) Confocal microscopic images of stained cells. Cells were fixed and stained with anti‐*γ*H2AX (green) and anti‐CD63 antibodies (red), and nuclei were stained with DAPI (blue), with quantitative presentation of the fold‐changes of *γ*H2AX‐positive foci in cells. Scale bars = 30 µm. A total of 60 randomly selected cells from three independent experiments were analyzed. I) Cells were fixed and stained with SA‐*β*‐gal. Scale bars = 50 µm. SA‐*β*‐gal‐positive cells were quantified. A total of 60 randomly selected cells from three independent experiments were analyzed. J–L) Fourteen‐month‐old mice (*n* = 6) were administered rapamycin (2.5 mg/kg/2d) or drug vehicle for 5 months. (J) Plasma exosomes were isolated from the treated animals and subjected to NTA. Six independent experiments were analyzed. (K) Tissues were collected, fixed, and stained with the anti‐*γ*H2AX antibody and (L) SA‐*β*‐gal. Scale bars = 50 µm. Relative positive cells were quantified. Three independent experiments were analyzed. *p < 0.05.

We further assessed the effects of mTORC1 activation on exosome release and aging progression. We generated tamoxifen‐induced systemic *TSC1* (mTORC1 upstream inhibitor^[^ ^33^ ^]^) KO mice by crossing *TSC1^flox/flox^* mice with *Ubc‐creERT2* mice to produce mice with constitutively activated mTORC1 (Figure S6A,B, Supporting Information). As predicted, constitutively activated mTORC1 not only significantly reduced exosome release, but also lost its function as a nutrient/energy senor, thus attenuating the effect of fasting on exosome release in mice (**Figure** **6**A–C). Moreover, compared with wild‐type mice, *γ*H2AX and SA‐*β*‐gal expression levels were significantly enhanced in *TSC1* KO mice, indicating that activation of mTORC1 accelerated senescence in mice (Figure 6D,E). These results were confirmed by P16 staining of the major organs (Figure S6D, Supporting Information).

We isolated tamoxifen‐induced constitutively activated mTORC1 MEFs from *TSC1* KO mice. Cell senescence was induced by successive passaging (passage 8), as indicated by increased *γ*H2AX expression and SA‐*β*‐gal staining in line with increasing passage number (Figure S6E,F, Supporting Information). The addition of tamoxifen induced *TSC1* deletion and mTORC1 activation, and expedited cellular aging in *TSC1* KO but not wild‐type MEFs (Figure 6F,G). Importantly, rapamycin treatment inhibited mTORC1 activity in these MEFs, and therefore decreased *γ*H2AX expression and SA‐*β*‐gal staining (Figure 6H,I). Intermittent dietary restriction (food ad libitum every other day) for 5 months and rapamycin^[^ ^34^ ^]^ reversed the senescent phenotype induced by mTORC1 activation in *TSC1^+/−^* mice (Figure S7, Supporting Information). Given that the administration of tamoxifen to induce *TSC1* deletion in mice was lethal, *TSC1^+/−^* mice were employed for evaluation in this study.

We further investigated the role of mTORC1 in exosome release and subsequent senescence in 14‐month‐old mice. As expected, 5 months of rapamycin treatment significantly increased the numbers of exosomes in the plasma of aged mice (≈2.81‐fold) (Figure 6J). Inhibition of mTORC1 by rapamycin also decreased the expression of *γ*H2AX and SA‐*β*‐gal (Figure 6K,L), indicating that mTORC1 inhibition delayed senescence through increased release of exosomes.

Taken together, these results substantiated a critical role for mTORC1 in exosome release and concomitant senescence, both in vitro and in vivo. mTORC1 activation inhibited the release of exosomes and accelerated aging, while mTORC1 inactivation promoted exosome secretion and delayed aging.

### 2.5 Exosome Release Contributes to Nutrient Restriction‐ or Rapamycin‐Delayed Aging

**Figure 7:**
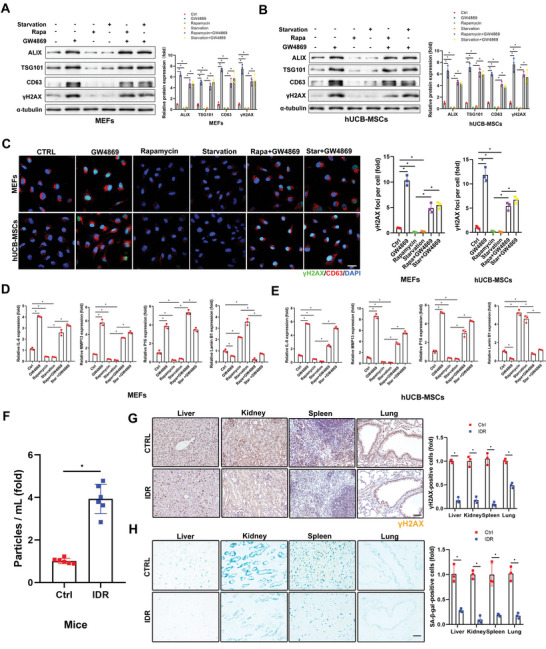
Increased exosome release contributes to nutrient restriction and rapamycin delayed aging. (A–F) MEFs and hUCB‐MSCs were continuously passaged until senescence and then treated with rapamycin (100 × 10^−9^ m) or cultured in 1% serum‐containing medium for 24 h before treating with GW4869 (2 × 10^−6^ m) for 5 d. A,B) Levels of exosome marker proteins and senescence marker proteins were determined by western blot with quantification. Three independent experiments were analyzed. C) Confocal microscopic images of stained cells. Cells were fixed and stained with anti‐*γ*H2AX (green) and anti‐CD63 antibodies (red), and nuclei were stained with DAPI (blue), with quantitative presentation of the fold‐changes of *γ*H2AX‐positive foci in cells. Scale bars = 30 µm. A total of 60 randomly selected cells from three independent experiments were analyzed. D,E) Expression levels of *IL6, MMP13, P16*, and *Lamin B1* mRNA were determined by qRT‐PCR. *GAPDH* was used as the internal control. Three independent experiments were analyzed. F–H) Fourteen‐month‐old mice (*n* = 6) were fasted intermittently for 5 months. (F) Plasma exosomes were isolated and subjected to NTA. Six independent experiments were analyzed. (G) The mice were sacrificed and tissues were collected, fixed, and stained with the anti‐*γ*H2AX antibody and (H) SA‐*β*‐gal. Scale bars = 50 µm. Relative positive cells were quantified. Three independent experiments were analyzed. *p < 0.05.

We next investigated the relationships between nutrient restriction‐ and mTORC1 inactivation‐stimulated exosome release and delayed senescence. Cell senescence was induced by successive passaging, and GW4869 increased, while serum deprivation and rapamycin treatment reduced CD63‐positive vesicles inside cells (**Figure** **7**A,B; Figure S8A, Supporting Information). Rapamycin‐ and serum deprivation‐stimulated exosome secretion were blocked by GW4869 (Figure 7C; Figure S8B,C, Supporting Information). We further assessed *γ*H2AX expression and mRNA expression levels of *IL6, MMP13, P16*, and *lamin B1* in all three types of cells following serum deprivation or rapamycin treatment with/without GW4869 treatment. Suppression of exosome release by GW4869 elevated all the above senescence markers (except for *lamin B1*, which was down‐regulated) in control cells, and reversed serum deprivation‐ and rapamycin treatment‐reduced senescence markers (except for *lamin B1*, which was up‐regulated) in all three different cell types (Figure 7D,E; Figure S8D, Supporting Information). Notably, GW4869 failed to completely block serum deprivation‐ and rapamycin‐reduced senescence biomarkers, implicating the involvement of an exosome secretion‐independent mechanism in this process. Altogether, these results suggest that serum deprivation or rapamycin delayed cell senescence partially via exosome release.

To verify the role of exosome release in nutrient restriction‐ and rapamycin‐delayed senescence in vivo, aged mice (14‐months‐old) were subjected to dietary restriction or rapamycin treatment. The number of extracted exosomes was significantly increased by ≈3.87‐fold in aged mice under IDR (intermittent dietary restriction, food ad libitum every other day) for 5 months compared with the control group (Figure 7F). Moreover, *γ*H2AX and SA‐*β*‐gal expression levels in the cells of major organs were markedly decreased in mice with IDR compared with control mice (Figure 7G,H). More CD63‐positive cells were observed in older compared with younger mice (Figure S9A, Supporting Information), indicating that exosome secretion was severely restricted in senescent mice. IDR and rapamycin treatment did not affect the body weight or microstructures of the major organs in older mice, but markedly delayed aging, indicated by increased lamin B1 staining, through promoting exosome secretion (Figure S9, Supporting Information). These findings thus indicated that exosome release was critical for nutrient restriction‐ or rapamycin‐delayed senescence in mice.

## 3 Discussion

**Figure 8:**
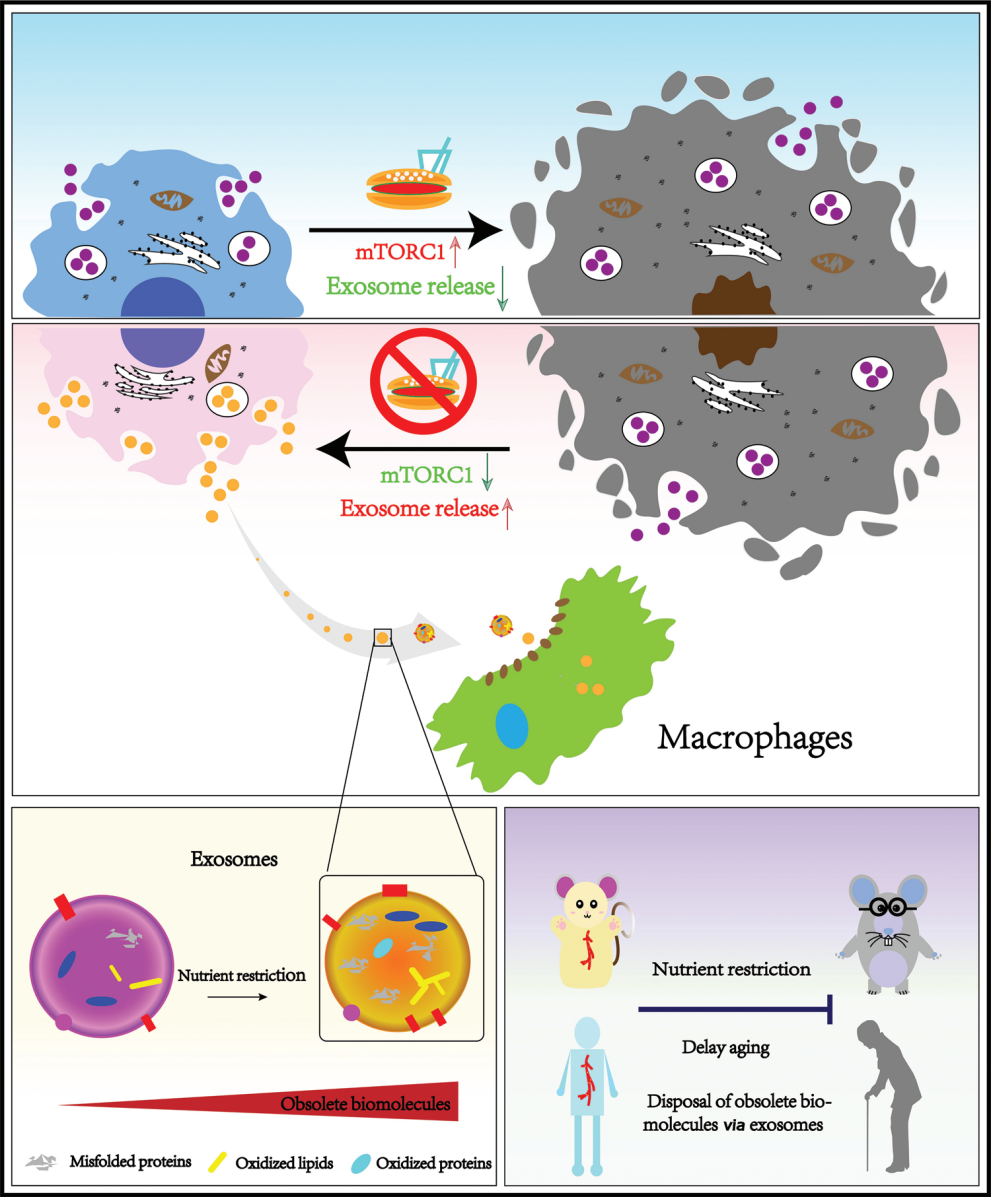
Exosome release delays senescence by disposing of obsolete biomolecules. Exosome release from cells was closely correlated with a senescent phenotype both in vitro and in vivo. Inhibition of exosome release accelerated, while stimulation of exosome secretion delayed cell senescence and organism aging. Increased exosome secretion by nutrient restriction or rapamycin allows the excretion of harmful biomolecules which can be disposed of by professional “cleaners”, such as macrophages.

In the present study, we demonstrated for the first time that exosome release from cells was closely correlated with a senescent phenotype both in vitro and in vivo. Inhibition of exosome release accelerated, while stimulation of exosome secretion delayed cell senescence and organism aging, including muscle tissues (skeletal, cardiac) (Figure S10, Supporting Information). Importantly, we found that increased exosome secretion by nutrient restriction or rapamycin allows the excretion of harmful biomolecules which accelerate cell senescence at least in vitro, and can be disposed of by professional “cleaners”, such as macrophages. Our findings thus established exosome release as an alternative and essential waste‐disposing pathway, representing a novel antiaging mechanism in multicellular organisms (**Figure** **8**).

Exosomes were first reported about four decades ago.^[^ ^35^ ^]^ Although exosomes have attracted extensive attention in terms of their prominent roles in cell communication,^[^ ^13^ ^]^ and their potential clinical applications,^[^ ^36^ ^]^ the biological function of exosomes release has not been fully elucidated. Recent studies indicate that exosomes contain mRNA, miRNA, DNA, proteins, and lipids.^[^ ^17^ ^]^ Interestingly, a report has shown harmful cytoplasmic DNA was loaded in exosomes.^[^ ^37^ ^]^ We showed that misfolded proteins, oxidative adducts and damaged DNA were encapsulated in exosomes (Figure 3). Metabolomics (Figure S11, Supporting Information) and proteomics (Figure S12, Supporting Information) on exosomes isolated from serum‐containing and serum‐deprived media of old MEFs (passage 8) revealed that the metabolomic profile of exosomes from starvation group was significantly different from that of the control group, and differential proteins were mainly enriched in nucleic acid metabolic process. Lysophosphatidylcholine (LPC) is a harmful metabolite of lipids and is associated with inflammation^[^ ^38^ ^]^ and oxidative stress.^[^ ^39^ ^]^ This is a representative obsolete molecule highly accumulated in the exosomes expelled by the starvation old cells. Confined these exosomes within cells accelerate senescence, but the stimulated release of exosomes can delay aging by disposing of these obsolete molecules at the cellular level in vitro, as well as at the organ and organism level in vivo. Exosomes isolated from cells or human plasma after nutrient restriction and rapamycin significantly accelerated mouse and human cell senescence, indicating that age‐promoting biomolecules were removed from normal cells to maintain homeostasis. Further studies are needed to determine if exosomes collected after fasting can accelerate aging in vivo. Altogether, our findings reveal an essential function of exosome release in garbage disposal, to maintain homeostasis and delay aging in multicellular organisms.

Robust and replicable advantageous effects of nutrient restriction on aging and lifespan have been well documented in lower eukaryotes and animal models.^[^ ^40^ ^]^ Although there is thus a basic consensus that nutrient restriction has the potential to delay aging and extend longevity in humans, the specific mechanism and beneficial effects of nutrient restriction in terms of metabolic switching and cellular stress resistance remain unclear.^[^ ^41^ ^]^ Nutrient restriction boosts mitochondrial function, stress resistance, and antioxidant defenses by activating various signaling pathways, and simultaneously enhances autophagy to remove damaged molecules and recycle their components.^[^ ^42^ ^]^ It has been well‐documented that inhibition of mTORC1 signaling by nutrient restriction or rapamycin delays aging through activating autophagy, and decreasing ER stress and ROS production.^[^ ^11^ ^]^ However, the current study uncovered an alternative mechanism in which inhibition of mTORC1 delayed aging in multicellular organisms by stimulating exosome release to expel damaged molecules for disposal. Convincing results from *TSC1* conditional KO mice and MEFs showed that constitutively activation of mTORC1 restricted the release of exosomes and accelerated senescence. Importantly, inhibition of exosome release partially reversed the promoted exosome secretion and delayed senescence resulted from mTORC1 inhibition. While the precise mechanisms underlying the obsolete molecules loading into exosomes requires further clarification, our results support that stimulated exosome release can delay aging, which was mediated by inhibition of mTORC1.

E3 ubiquitin ligases are mainly responsible for labeling proteins that are ubiquitinated in a dynamic and tightly regulated manner; however, this process can be reversed by deubiquitinases.^[^ ^43^ ^]^ The current results showed that, after fasting, exosomes contained more deubiquitinases, such as USP5, USP24, UCHL1, UCHL5, inferring that cells discharged these molecules to maintain the efficiency of the ubiquitin‐proteasome system. In addition, misfolded proteins, oxidized adducts were expelled by cells through the release of exosomes after nutrient restriction, indicating a possible alternative route for handling damaged proteins, especially when the two classic systems may be overloaded. Our results showed that in the multicellular organism, the exosomes containing garbage molecules processed by the “cleaners”, namely macrophages, was one coordinative way. Consistently, a resent research demonstrated that macrophages lodged within the healthy myocardium actively took up wastes expelled by the cardiomyocytes to help maintain heart homeostasis.^[^ ^44^ ^]^ These evidences further clarify the regime of multicellular organism orchestrates garbage disposal and processing to delay aging. Furthermore, multiple age‐related diseases, including neurodegeneration,^[^ ^45^ ^]^ diabetes,^[^ ^46^ ^]^ cystic fibrosis,^[^ ^47^ ^]^ and cardiac amyloidosis,^[^ ^48^ ^]^ mainly or partially result from the inability of cells to degrade misfolded or damaged proteins. Our findings may thus shed a light on alternative strategies for expelling damaged proteins via exosomes and macrophages to ameliorate and treat those diseases.

In summary, our findings support a model in which nutrient restriction stimulated exosome release delays cell senescence in vitro and aging of multicellular organisms in vivo by removing obsolete biomolecules, including misfolded proteins and oxidized adducts, as well as damaged DNA fragments in cells, mice, and humans. These results highlight an important role of exosome secretion, and provide additional evidence in relation to fasting‐delayed aging, thus providing a basis for further investigations on multicellular organisms orchestrating waste processing.

## 4 Experimental Section

MEFs and hUCB‐MSCs were primarily cultured from the newborn placenta and mouse embryos. *Rab27^−/−^* and conditional *TSC1* ^+/−^ mice were generated and animal experiments were approved by the Ethics Committee for Animal Research of Southern Medical University. Human volunteers were recruited with written consent forms and the project was approved by the medical ethics review committee at the Third Affiliated Hospital of Southern Medical University. The detailed Experimental Section for the cell cultures, exosomes and corresponding analysis, WB, RT‐qPCR, histology and immunohistochemistry, and statistical analysis can be found in the Supporting Information.

## Conflict of Interest

The authors declare no conflict of interest.

## Author Contributions

X.B. and Z.‐K. C. conceived, designed the project, supervised experiments, wrote and revised the manuscript. W.Z., M. L., Y.J., and L.M., collected the data. W.Z., S.Z., P.L, B.G., T.W., C.N., L.Z., S.L., A.L., and J.Z., contributed to data and analysis. L.X. and Z.Z. collected clinical samples. All authors approved the final version of the manuscript.

## Significance Statement

Substantial evidence has highlighted the beneficial effects of nutrient restriction in delaying cellular senescence and organism aging, particularly in lower eukaryotes and animal models; however, the influence of nutrient restriction on human aging and longevity, as well as its underlying mechanisms, remain debatable. Inhibition of exosome release either in vitro or in mice accelerated senescence. An alternative mechanism was proposed, by which nutrient restriction delays aging in multicellular organisms by orchestrating the disposal of obsolete biomolecules via exosomes and macrophages.

## Supporting information

Supporting Information

Supporting Information

## Data Availability

Detailed information on the antibodies, biological samples, critical commercial assays, and oligonucleotides is listed in the Supporting Information. Further information and requests for reagents should be directed to and will be fulfilled by Zhong‐Kai Cui (zhongkaicui@smu.edu.cn) and Xiaochun Bai (baixc15@smu.edu.cn). Also, the data that support the findings of this study are available from the corresponding authors upon reasonable request.
