# Supplementary material for: Exosome Release Delays Senescence by Disposing of Obsolete Biomolecules
Source: Adv Sci (Weinh). 2023 Jan 22;10(8):2204826. doi: 10.1002/advs.202204826 (PMC10015858; doi:10.1002/advs.202204826)
Supplement: Supplementary file 1 — Supporting Information [file ADVS-10-2204826-s002.pdf]

Supporting Information for

Exosome release delays senescence by disposing of obsolete biomolecules

Wenchong Zou[1,2,#], Mingqiang Lai[3,#], Yuanjun Jiang[2,#], Linlin Mao[2], Wu Zhou[2], Sheng Zhang[2], Pinglin Lai[1], Bin Guo[2], Tiantian Wei[2], Chengtao Nie[4], Lei Zheng[4], Jiahuan Zhang[2], Xuefei Gao[5], Xiaoyang Zhao[6], Laixin Xia[6], Zhipeng Zou[2], Anling Liu[7], Shiming Liu[8], Zhong-Kai Cui[2,9,*], Xiaochun Bai[1,2,*]

# These authors contributed equally to this work.

* To whom correspondence may be addressed. Email: Dr. Xiaochun Bai (Email: baixc15@smu.edu.cn) and Dr. Zhong-Kai Cui (Email: zhongkaicui@smu.edu.cn)

This file includes:

SI Materials and Methods

Materials and Resources Table

Figs. S1-S12

SI References

SI Materials and Methods

Human participants

This project recruited volunteers from the Third Affiliated Hospital of Southern Medical University (Guangzhou, China), including 16 healthy elderly (60–70 years old) and young people (20–30 years old). Whole blood samples were obtained during a normal diet. Control blood samples were obtained after eating normally. The volunteers were allowed to drink water freely. Further blood samples were collected after fasting for 24 h (Fasting), and 24 h after the recovery of the diet (Recovery group). The project was approved by medical ethics review at the Third Affiliated Hospital of Southern Medical University (all the participants provided written informed consent.).

Mice

Animal experiments were approved by the Ethics Committee for Animal Research of Southern Medical University and conducted according to the state guidelines from the Ministry of Science and Technology of China. *TSC1* flox/flox (Stock No: 005680) and UBC-cre/ERT2 (Stock No: 008085) mice were purchased from the Jackson Laboratory (Las Vegas, NV, USA) and crossed to generate inducible *TSC1*-deletion mice. To induce *TSC1* recombination, male mice of these genotypes were injected with 100 μl tamoxifen solution intraperitoneally for 8 consecutive days. Tamoxifen (Sigma-Aldrich, St. Louis, MO, USA) was dissolved in sterilized corn oil for 24 h at a concentration of 20 mg/mL. The single-guide RNA (sgRNA) target site was designed according to the *Rab27a* coding region, and *in vitro* transcription of sgRNA and Cas9 mRNA was performed. Three-week-old C57BL/6 female mice were subjected to superovulation and then caged with C57BL/6 male mice of the appropriate age. The next day, fertilized eggs were collected after checking vaginal plugs, and sgRNA and Cas9 mRNA were microinjected into the oviducts of surrogate mothers. The PCR

amplification primers were designed according to the KO sequence, and the genotypes of the F0 generation mice were identified. *Rab27a* KO mice had grayer coats than wild-type mice. All lines were backcrossed to the C57BL/6J background. C57BL/6 mice (newborn or 6–7 weeks old) were purchased from the Laboratory Animal Center of Southern Medical University (Guangzhou, China). The exosome inhibitor GW4869 (Sigma-Aldrich) was injected into the tail vein after placing the mouse in a fixer, straightening the tail, and wiping it with an alcohol cotton ball. The tail was then held with the left hand and the syringe with the right hand. The needle was inserted slowly until the blood returned, and the solution was then injected slowly. GW4869 was dissolved in dimethylsulfoxide (DMSO) at 8 mg/mL. The working solution was freshly prepared before use in 0.9% normal saline, with a final concentration of 0.3 mg/mL (2.5 μg/g body weight). For intermittent dietary restriction in elderly mice (14 months old), the mice were allowed to drink and eat freely every other day for 5 months. Mice were bred and maintained under specific pathogen-free conditions in a temperature- and humidity-controlled environment with a 12 h light/dark cycle (06:00–18:00).

Isolation of primary cells

Isolation of hUCB-MSCs: hUCB-MSCs were isolated from newborn placenta according to a previously described method (Kern et al., 2006). Briefly, prior to the isolation of multinucleated cells, anticoagulated cord blood was diluted 1:1 with 2 mM EDTA-PBS. The isolated cells were then seeded at a density 2×10^5 cells per cm^2 on plates in growth medium containing D-media (Formula 78-5470EF; Gibco, Carlsbad, CA, USA), the endothelial cell growth medium-2 (EGM-2), and 10% FBS (Gibco). Adherent fibroblastoid cells which appeared as colony forming unit-fibroblastic (CFU-F) were then harvested at subconfluence using trypsin (Gibco) and sub-cultured(1).

Differentiating macrophages from bone marrow cells: Mice were euthanized by CO_2 inhalation, and death was ensured by cervical dislocation. The femur was separated

from the tibia by dislocation and cutting through the knee joint, the tibia was separated from the foot by cutting just below the ankle joint, and tissue was removed from the bones. The epiphyses of the bones were cut and the marrow was flushed into a 50 mL centrifuge tube using a 5 mL syringe and a 23 G needle, with 5 mL of phosphate-buffered saline (PBS) per bone. The cell suspension was then filtered through a 70 μm cell strainer into a new 50 mL tube and centrifuged for 5 min at 250 × *g* and 4°C. The supernatant was discarded and the cell pellet was resuspended in 3 mL of bone marrow-derived macrophages (BMDM) medium. Twenty microliters of the suspension was diluted in 180 μL PBS, and 20 μL of this dilution was added to 80 μL of 2% acetic acid, and incubated for 2–3 min at room temperature. The cells were counted under a microscope using a hemocytometer. Seven milliliters of BMDM medium was then added to the non-diluted cells and 10 mL of suspension was seeded on a 10 cm cell culture-treated petri dish and incubated for 3 h at 37°C with 5% CO_2. The cells were then diluted to a concentration of 3.5×10^5 cells/mL, macrophage colony-stimulating factor (R&D Systems, Minneapolis, MN, USA) was added at a final concentration of 25 ng/mL, and 10 mL of cells was seeded per 10 cm cell culture-treated petri dish and incubated at 37°C with 5% CO_2 for 3 days. The medium was then replaced for further incubation(2).

Isolation of MEFs from mouse embryos: Pregnant female mice were anesthetized at day E14.5 by intraperitoneal injection of 0.5 mL 2.5% avertin, and then euthanized by cervical dislocation. A small incision was cut through the skin at the centric position on the middle abdomen. The embryos were removed and placed in a sterile dish, and 3 mL of ice-cold 0.25% trypsin-EDTA was added to the dish. Two pairs of fine forceps were then used to tease the fetuses into fine pieces. All the material was transferred to a 15-mL tube (for up to four embryos) using a pipette, and ice-cold 0.25% trypsin-EDTA was added to bring the total volume to 3 mL per embryo. The tube was allowed to stand overnight at 4°C, and was then incubated for 30 min in a 37°C water bath. MEF culture medium was added to 8 mL (per 15-mL tube) and pipetted vigorously and repeatedly up and down to break up the digested tissues into a

cell suspension. The cell suspension was allowed to settle for 1 min to let any remaining clumps of tissue fall to the bottom of the tube. The supernatant cell suspension was then transferred to a new tube and mixed, and the cells were plated in 10-cm tissue culture dishes with 10 mL MEF culture medium(3).

Cell culture

MEFs and HUVECs were cultured in high-sugar Dulbecco's Modified Eagle's Medium (DMEM, Gibco) containing 10% FBS. hUCB-MSCs were inoculated into 10% FBS, 1% sodium pyruvate, L-glutamine, MEM non-essential amino acids, low-sugar DMEM medium (all from Gibco) and placed in an incubator, and subcultured at ratios of 1:3 or 1:4. GW4869 powder 2 mM was dissolved in DMSO (Sigma-Aldrich) and sterilized by a 220-nm filter. Rapamycin (MCE, Monmouth Junction, NJ, USA) was dissolved in DMSO (Sigma-Aldrich), sterilized by a 220-nm filter, and 100 nM was added to the culture medium. All cells were cultured in a 37°C incubator with 5% CO_2 and 95% humidity. The culture medium was replaced every other day, or according to experimental requirements.

Isolation of exosomes

The culture medium (depleted from serum exosomes) was collected and centrifuged at 320 × *g* for 10 min to pellet the cells, followed by 2,000 × *g* for 10 min to remove cell debris and dead cells, and 10,000 × *g* for 30 min at 4°C. The medium was further centrifuged at 100,000 × *g* for 70 min at 4°C (Beckman Coulter Optima L-100 XP, Beckman Coulter). The precipitate was washed twice with PBS, centrifuged at 100,000 × *g* for 70 min, and the exosomes at the bottom of the tube were collected. Whole blood was drawn into an EDTA anticoagulation tube and centrifuged to obtain separated plasma. Samples were centrifuged at 3000 × *g* for 15 min at 4°C and transferred to a new 1.5 mL centrifuge tube, followed by centrifuging at 10,000 × *g* for 30 min at 4°C. The supernatant was then diluted with PBS buffer and transferred

to a Pollyallomer high-speed centrifuge tube (Sigma-Aldrich) and centrifuged at 100,000 × *g* for 70 min at 4°C using a ultracentrifuge (Beckman). The pellet was washed twice with PBS and centrifuged at 100,000 × *g* for 70 min to collect the exosomes at the bottom of the tube, before resuspension in RIPA buffer (Sigma-Aldrich). Protein concentration was determined by BCA Protein Assay (Abcam, Cambridge, UK).

Density-gradient centrifugation

Optiprep stock solution (Axis-Shield, Dundee, Scotland, UK) was diluted 60% w/v with Tris-Sucrose buffer. Three milliliters of 40%, 20%, and 10% (w/v) OptiPrepiodixanol solution (Axis-Shield) and 2.5 mL of 5% iodixanol solution were layered sequentially in ultracentrifuge tubes, and 500 μL of the isolated exosomes were overlaid on a discontinuous gradient and centrifuged at 110,000 × *g* for 18 h. Following centrifugation, 1 mL fractions were collected from top to bottom, centrifuged at 100,000 × *g* for 1 h at 4°C, and resuspended in 50–80 μL PBS.

Nanosight analysis

The exosomal samples were diluted and the concentration was adjusted as required (1×10^8–1×10^{11}/mL) for characterization. The number and size distribution of the exosomes were detected using a Nanosight N3000 nanoparticle analyzer (Malvern, UK), and the data were analyzed using the related software NTA 3.2 Dev Build 3.2.16 (Malvern).

Protein extraction and western blotting

For total protein extraction, cells, exosomes, and tissues were lysed in RIPA buffer containing 50 mM Tris-HCl pH8, 150 mM NaCl, 1% Triton X-100, 0.1% sodium deoxycholate, 0.1% sodium dodecylsulfate (SDS), and 1× protease inhibitor cocktail

(Roche, Basel, Switzerland). Lysates were boiled in 2× SDS sample buffer for 10 min and proteins were then separated by SDS-polyacrylamide gel electrophoresis and transferred to nitrocellulose membranes (Bio-Rad, CA, USA) for immunoblotting blocking treatment and incubation with CD63 (Abcam, Cat#ab217345, RRID: AB_2754982), Alix (Proteintech, Cat#12422-1-AP, RRID: AB_2162467), H3K9me3 (Abcam, Cat#ab8898, RRID: AB_306848), Phospho-Histone H2A.X (Abcam, Cat#ab217345, RRID: AB_2754982), pS6 (Cell Signaling Technology, Cat#2211S, RRID：AB_331679), RAB27A (Proteintech, Cat#17817-1-AP, RRID：AB_2176728), S6 (Santa Cruz, Cat#sc-74459, RRID: AB_1129205), TSC1 (ABclonal, Cat# A0720, RRID: AB_2757364), TSC2 (Cell Signaling Technology, Cat#4308T, RRID： AB_10547134), TSG101 (Proteintech, Cat#14497-1-AP, RRID： AB_2208090), Ubiquitin (Proteintech, Cat#10201-2-AP, RRID: AB_671515), USP24 (Proteintech, Cat#13126-1-AP, RRID: AB_2212761), α-tubulin (Beijing Ray Antibody Biotech, Cat#RM2007) overnight at 4°C. Chemiluminescence imaging was performed using a Western Lightning Plus ECL kit (Perkin Elmer, MA, USA).

SA-β-gal assay

Senescent cells were stained using an SA-β-gal staining kit (Cell Signaling Technology, Cat#9860S, Boston, MA, USA). MEFs, HUVECs, and hUCB-MSCs in 12-well plates, and frozen tissue section were fixed with 4% paraformaldehyde for 10 min at room temperature and stained overnight at 37°C. The staining solution was then removed and the samples were washed in PBS. Senescent cells, identified as blue-stained cells, were detected by light microscopy (Nikon TE2000-U; Zeiss Axio Scope.A1, Carl Zeiss, Oberkohen, baden-Wurberg, Germany).

Frozen tissue sections were detected using an SA-β-gal staining kit (Beyotime, Jiangsu, China). Mice were euthanized and tissues were fixed in 4% paraformaldehyde for 24 h, followed by transfer to 30% sucrose for 24 h for dehydration. Sagittal sections (5 μm) were prepared using a Leica CM1850 cryostat (Leica, Wetzlar, Germany) and incubated for 15 min at 37°C to remove opti-mum

cutting temperature compound (OCT) embedding medium, and then stained using SA-β-gal staining.

Immunohistochemistry

Immunohistochemistry was performed on 4 μm sections of formalin-fixed paraffin-embedded tissues. The sections were dehydrated and deparaffinized using xylene and graded ethanols, and then soaked in citrate buffer (10 mM citric acid, pH6.0) for 16 h at 60°C to unmask antigens. After rewarming and washing with PBS, 3% hydrogen peroxide solution was added dropwise and incubated at room temperature for 10 min. The sections were then blocked with 1% goat serum at 37°C for 1 h, stained with CD63 (Abcam, Cat#ab193349), p16 (Santa Cruz, Cat#sc-1661, RRID: AB_628067), Phospho-Histone H2A.X (Abcam, Cat#ab217345, RRID: AB_2754982), pS6 (Cell Signaling Technology, Cat#2211S, RRID: AB_331679), Lamin B1 (Proteintech, Cat#14497-1-AP, RRID：AB_2208090) overnight at 4°C, and then with the relevant secondary antibody for 1 h at 37°C. Finally, the color was developed with DAB (ZSGB-BIO, ZLI-9018, Beijing, China) and the nuclei were counterstained with hematoxylin, and the slides were mounted with neutral resin. Stained sections were imaged on an Axio Scope A1 microscope (Carl Zeiss).

Immunofluorescence

Cells were fixed with 4% paraformaldehyde at room temperature for 15 min and stained with the indicated primary antibodies that recognized CD63 (Abcam, Cat#ab193349), Phospho-Histone H2A.X (Abcam, Cat#ab217345, RRID: AB_2754982), and F4/80 (Abcam, Cat#ab6640, RRID: AB_1140040) overnight at 4°C, followed by fluorescent-dye-conjugated secondary antibodies (ThermoFisher, Waltham, MA, USA) for 1 h at 37°C. Nuclei were stained with DAPI (Sigma-Aldrich). Images were obtained using a confocal laser scanning microscope (Olympus FV1000, Tokyo, Japan) and processed using FV10-ASW 3.1 software

(Olympus).

Real-time RT-PCR

Cells were collected and washed twice with cold PBS (pH7.4). Total RNA was extracted using Trizol plus RNA purification kit (Takara Bio, Japan). Total RNA (0.2–1 μg) was reverse transcribed into cDNA using a PrimeScript RT kit (Vazyme Biotech, Nanjing, China) and RT-PCR was carried out using SYBR Premix Ex Taq II (Vazyme Biotech). Relative quantification was calculated using the $2^{-\Delta\Delta ct}$ method. All data were normalized against endogenous GAPDH controls of each sample.

Cell Counting Kit-8 (CCK-8) assay

The CCK-8 assay (MCE, Monmouth Junction, NJ, USA) was used to determine cell viability. A total of 2×10^3 cells (100 μL/well) were seeded in 96-well plates and allowed to adhere for 24 h, followed by the addition of 10 μL of CCK-8 solution to each well (being careful not to generate air bubbles). The cells were incubated for 2 h at 37°C and the absorbance was measured at 450 nm using a microplate reader (Synergy HTX Multi-Mode Reader, BioTek, Santa Clara, CA, USA) .

In vivo imaging and tissue distribution

Plasma was obtained from fasting mice and the exosomes were extracted by ultracentrifugation and incubated with DiD (a hydrophobic dye; AAT Bioquest, CA, USA) at room temperature for 10 min. The exosomes (50 μg/mL) were then injected into the tail vein of mice. The mice were anesthetized with pentobarbital (35–40 mg/kg). The comatose mice were shaved and the distribution of the exosomes was investigated using a near-infrared *in vivo* imaging system (FX Pro, Bruker, Madison, WI, USA). After 6 h, the mice were euthanized and their organs were imaged and tested.

Zetasizer nanoanalysis

Protein samples were lysed in 100 µL RIPA buffer (Sigma-Aldrich). The granularity of the particles was measured using a nanosizer (Malvern, UK), according to the manufacturer's instructions, with a conductivity value of 0.093 mS/cm and a voltage of 3.9 V.W.

Circular dichroism

Exosomes or cell suspension 100 µL of sonication (50 w 1s on, 2 s off, 2 min). After sonication, centrifuge at 12,000 × g, 10 min, 4°C, remove the supernatant and dilute to 300 µL with PBS. The circular dichroism instrument (Chirascan, Applied Photophysics Ltd, UK) was turned on and nitrogen gas was purged for at least 20 min. Set parameters to temperature 4°C; wavelength 180–260 nm; then add 300 µL of sample to the cuvette for detection.

Quantification and statistical analysis

Quantitation was performed using Image J software (NIH, Bethesda, MD, USA). Statistical analyses were carried out using GraphPad Prism 6.0 software (GraphPad Prism, San Diego, CA, USA). Differences between two groups were analyzed by two-tailed unpaired Student's *t*-test. A p value < 0.05 was considered statistically significant. All experiments were repeated at least three times. Quantitative data were expressed as mean ± standard deviation. Statistical analysis used in each panel was described in the figure legends.

Materials and Resources Table

Detailed information on the antibodies, biological samples, critical commercial assays, and oligonucleotides

Reagent or Resource	Source	Identifier
**Antibodies**		
Alix Rabbit Polyclonal Antibody	Proteintech	Cat#12422-1-AP; RRID：AB_2162467
Anti-CD63 Rabbit antibody [EPR21151]	abcam	Cat#ab217345; RRID：AB_2754982
Anti-CD63 Mouse antibody [MX-49.129.5]	abcam	Cat#ab193349
Anti-F4/80 antibody	abcam	Cat#ab6640; RRID：AB_1140040
α-tubulin Mouse Monoclonal Antibody	Beijing Ray Antibody Biotech	Cat#RM2007
H3K9me3Antibody	abcam	Cat#ab8898; RRID：AB_306848
Lamin B1 Rabbit Polyclonal Antibody	Proteintech	Cat#12987-1-AP; RRID：AB_2136290
M594（ALEXA FLUOR 594 DONKEY）	ThermoFisher	Cat#A21203; RRID：AB_141633
M488（ALEXA FLUOR 488 DONKEY）	ThermoFisher	Cat#A21202; RRID：AB_141607
P16-INK4A Rabbit Polyclonal	Proteintech	Cat#10883-1-AP;

Antibody		RRID：AB_2078303
p16 Antibody	Santa Cruz Blotechnology	Cat#sc-1661; RRID：AB_628067
Phospho-Histone H2A.X (Ser139) (20E3) Rabbit mAb	Cell Signaling Technology	Cat#9718S; RRID：AB_2118009
pS6 Ribosomal Protein (Ser235/236) Antibody	Cell Signaling Technology	Cat#2211S; RRID：AB_331679
Peroxidase-conjugated AffiniPure Goat Anti-Rabbit IgG (H+L)	Jackson	Cat#111-035-003; RRID：AB_2313567
Peroxidase-conjugated AffiniPure Goat Anti-Mouse IgG (H+L)	Jackson	Cat#115-035-003; RRID：AB_10015289
RAB27A Rabbit Polyclonal Antibody	Proteintech	Cat#17817-1-AP; RRID：AB_2176728
Ribosomal protein S6	Santa Cruz Blotechnology	Cat#sc-74459; RRID：AB_1129205
R594（ALEXA FLUOR 594 DONKEY）	ThermoFisher	Cat#A21207; RRID：AB_141637
R488（ALEXA FLUOR 488 DONKEY）	ThermoFisher	Cat#A21206; RRID：AB_2535792
TSC1 Rabbit pAb	ABclonal	Cat# A0720; RRID:AB_2757364
Tuberin/TSC2 (D93F12) XP	Cell Signaling	Cat#4308T;

Rabbit mAb	Technology	RRID：AB_10547134
TSG101 Rabbit Polyclonal Antibody	Proteintech	Cat#14497-1-AP; RRID：AB_2208090
ubiquitin Rabbit Polyclonal Antibody	Proteintech	Cat#10201-2-AP; RRID：AB_671515
USP24 Polyclonal Antibody	Proteintech	Cat# 13126-1-AP; RRID：AB_2212761
USP5 Polyclonal antibody	Proteintech	Cat# 10473-1-AP; RRID：AB_2272754
UCHL1 Polyclonal antibody	Proteintech	Cat# 14730-1-AP; RRID：AB_2210497
UCHL5 Polyclonal antibody	Proteintech	Cat# 11527-1-AP; RRID：AB_2877774
ATXN3 Polyclonal antibody	Proteintech	Cat# 13505-1-AP; RRID：AB_2061192
JAB1 Polyclonal antibody	Proteintech	Cat# 27511-1-AP; RRID：AB_2880894
**Biological samples**		
Human Plasma (20-30&60-70)	Third Affiliated Hospital of Southern	N/A

	Medical University	
**Chemicals, peptides, and recombinant proteins**		
Sodium Pyruvate (100 mM)	Gibco	Cat#11360070
L-Glutamine 200mM (100x)	Gibco	Cat#25030-081
MEM Non-Essential Amino Acids Solution(100x)	Gibco	Cat#11140-050
NotI enzyme	NEB	Cat#R0189S
Pen Strep	Gibco	Cat#15140-122
Complete Protease Inhibitor mini EASY packs EDTA-Free	Roche	Cat#05892791001
β -Mercaptoethanol	Sigma-Aldrich	Cat#444203
Tris-MOPS Running Buffer Powder(Native)	GenScript	Cat#No.M007271
PageRulerTMPrestained Protein Ladder	ThermoFisher	Cat#26616
RNAiso Plus TRIZOL	Takara	Cat#9109
Bovine Serum Albumin	Sigma-Aldrich	Cat#9048-46-8
Dimethyl sulfoxide	Sigma-Aldrich	Cat#D5879-100ML
Triton X-100	Yeasen	Cat#20107ES76
Tamoxifen	Sigma-Aldrich	Cat#T5648-1G

DiD labeling solution	AAT Bioquest	Cat#22033
Normal Goat Serum	BOSTER	Cat#AR0009
Foetal Bovine Serum	Biological Industries	Cat#04-400-1A
Rapamycin	MCE	Cat#HY-10219
Sirolimus solution	Supelco	Cat#S-015
Paraformaldehyde	Macklin	Cat#P804537
Phalloidin, Fluorescein Isothiocyanate Labeled	Sigma-Aldrich	Cat#P5282
DAPI	Sigma-Aldrich	Cat#D5942-1MG
rmM-CSF, CF (10 ug)	R&D Systems	Cat#416-ML-010/CF
RIPA Buffer	Sigma-Aldrich	Cat#R0278
GW4869	Sigma-Aldrich	Cat#567715
Trypsin	Sigma-Aldrich	Cat#T4174
DMEM Low glucose	Gibco	Cat#11885084
DMEM High glucose	Gibco	Cat#11965092
**Critical commercial assays**		
Senescence beta-Galactosidase Staining Kit	Cell Signaling Technology	Cat#9860S
Senescence beta-Galactosidase Staining Kit	Beyotime	Cat#C0602
BCA protein content detection kit	Abcam	Cat#ab102536

Cell Counting Kit-8	MCE	Cat#HY-K0301
MinElute PCR Purification kit	Qiagen	Cat#28004
MEGA shortscript T7 Transcription kit	Thermo	Cat#AM1354
mMESSAGE mMACHIN T7 ULTRA kit	Thermo	Cat#AMB13455
HiScript Q RT SuperMix for qPCR（+g DNA wiper）	Vazyme	Cat#R123-01
ChamQ® SYBR® qPCR Master Mix(High ROX Premixed)	Vazyme	Cat#Q341-03
DAB Kit	ZSGB-BIO	Cat#ZLI-9018
Western Lightning Plus ECL	Perkinelmer	Cat#0RT2655
Mouse malondialchehyche,MDA ELISA Kit	Dogesce	Cat#DG30429M
Human malondialchehyche,MDA ELISA Kit	Dogesce	Cat#DG10351H
Mouse 4-HNE ELISA Kit	Dogesce	Cat#DG94695Q
Human 4-HNE ELISA Kit	Dogesce	Cat#DG12355H
Human Advanced oxidation protei n products ELISA Kit	Mlbio	Cat#ml030481
Mouse Advanced oxidation protein produ cts ELISA Kit	Mlbio	Cat#ml024089

Clophosome® - Neutral Liposomal Clodronate for Macrophage Depletion	formumax scientific	Cat#F70101C-N
**Experimental models: Cells**		
MEFs	David J Kwiatkowski , Brigham and Women's Hospital,USA	N/A
TSC2-/- MEFs	David J Kwiatkowski , Brigham and Women's Hospital, USA	N/A
HUVECs	ATCC	Cat#PCS-100-013
**Experimental models: Organisms/strains**		
TSC1 flox/flox mice (C57BL/6J)	The Jackson Laboratory	Stock No: 005680
UBC-cre/ERT2 mice (C57BL/6J)	The Jackson Laboratory	Stock No: 008085
Rab27a KO mice (C57BL/6J)	This paper	N/A
C57BL/6J	Laboratory Animal Center	N/A

	of Southern Medical University	
Oligonucleotides		
UBC-Cre-ERT2 (F)-genotyping primer GACGTCACCCGTTCTGTTG	The Jackson Laboratory	N/A
UBC-Cre-ERT2 (R)-genotyping primer AGGCAAATTTTGGTGTACGG	The Jackson Laboratory	N/A
TSC1-Flox(F)-genotyping primer GTCACGACCGTAGGAGAAGC	The Jackson Laboratory	N/A
TSC1-Flox(R)-genotyping primer GAATCA ACCCCACAGAGCAT	The Jackson Laboratory	N/A
Rab27a Forward 1 AGCTGCGAGTGACCTTTCTATC	This paper	N/A
Rab27a Forward 2 CCTTGGCTTATCTTTGCTTTGCTG	This paper	N/A
Rab27a Reverse 1 GAGCCTGAAACGGGAGAAA	This paper	N/A

GAA		
TSC1 Forward<br>GTCACGACCGTAGGAGAAGC	This paper	N/A
TSC1 Reverse<br>GAATCA ACCCCACAGAGCAT	This paper	N/A
UBC Forward<br>GACGTCACCCGTTCTGTTG	This paper	N/A
UBC Reverse<br>AGGCAA ATT TTGGTGTACGG	This paper	N/A
IL-6 mice Forward<br>CCGGAGAGGAGACTTCACAG	This paper	N/A
IL-6 mice Reverse<br>TCCACGATTTCCCAGAGAAC	This paper	N/A
IL-6 human Forward<br>TACCCCCAGGAGAAGATTCC	This paper	N/A
IL-6 human Reverse<br>TTTTCTGCCAGTGCCTCTTT	This paper	N/A
P16 mice Forward<br>GAAGCCGGGGTTTCGCCCAA	This paper	N/A
P16 mice Reverse<br>GCACCGGGCGGGAGAAGGTA	This paper	N/A
P16 human Forward	This paper	N/A

AGCTGGAATTACACAGCTGC		
P16 human Reverse<br>GGACTGGCTTGCAATCTTGT	This paper	N/A
MMP13 mice Forward<br>GGAGCCCTGATGTTTCCCAT	This paper	N/A
MMP13 mice Reverse<br>GTCTTCATCGCCTGGACCATA	This paper	N/A
MMP13 human Forward:<br>CACTCACAGACCTGACTCGG	This paper	N/A
MMP13 human Reverse<br>GAGTCAGGGGGAGGTCCATA	This paper	N/A
Lamin B1 mice Forward<br>GGGAAGTTTATTCGCTTGAAG<br>A	This paper	N/A
Lamin B1 mice Reverse<br>ATCTCCCAGCCTCCCATT	This paper	N/A
Lamin B1 human Forward<br>AAGCAGCTGGAGTGGTTGTT	This paper	N/A
Lamin B1 human Reverse<br>TTGGATGCTCTTGGGGTTC	This paper	N/A
GAPDH mice Forward<br>AGGTCGGTGTGAACGGATTT	This paper	N/A

G		
GAPDH mice Reverse GGGGTCGTTGATGGCAACA	This paper	N/A
GAPDH human Forward CAACTTTGGCATTGTGGAAG G	This paper	N/A
GAPDH human Reverse ACACATTGGGG GTAGGAACAC	This paper	N/A
Rab27a-sgRNA1 GTCTAAACCTGGCTCTGTTC	This paper	N/A
Rab27a-sgRNA2 CACACTGACCTGGGCAGGAG	This paper	N/A
Rab27a-sgRNA3 GAAAGTCAAGAAGAGCCCGC	This paper	N/A
Rab27a-sgRNA4 GGCCAGGCAAAGGCAGCCA	This paper	N/A
**Software and algorithms**		
Image J	Image J	https://imagej.nih.gov/ij/
FV10-ASW 3.1	Olympus	http://www.olympus.com.cn/
ZEN 2011	Zeiss	https://www.zeiss.com
Imaris 8.1	Nikon	https://www.nikon.com.cn/s

		c_CN/
GraphPad Prism 6.0	Prism	http://www.graphpad-prism.cn/prism.html
Adobe Photoshop 6.0	Adobe	https://www.adobe.com/cn/products/photoshop.html
Bruker MI SE 7.2	Bruker	https://www.bruker.com/zh.html
NTA 3.2 Dev Build 3.2.16	Malvern	https://www.malvernpanalytical.com/en/

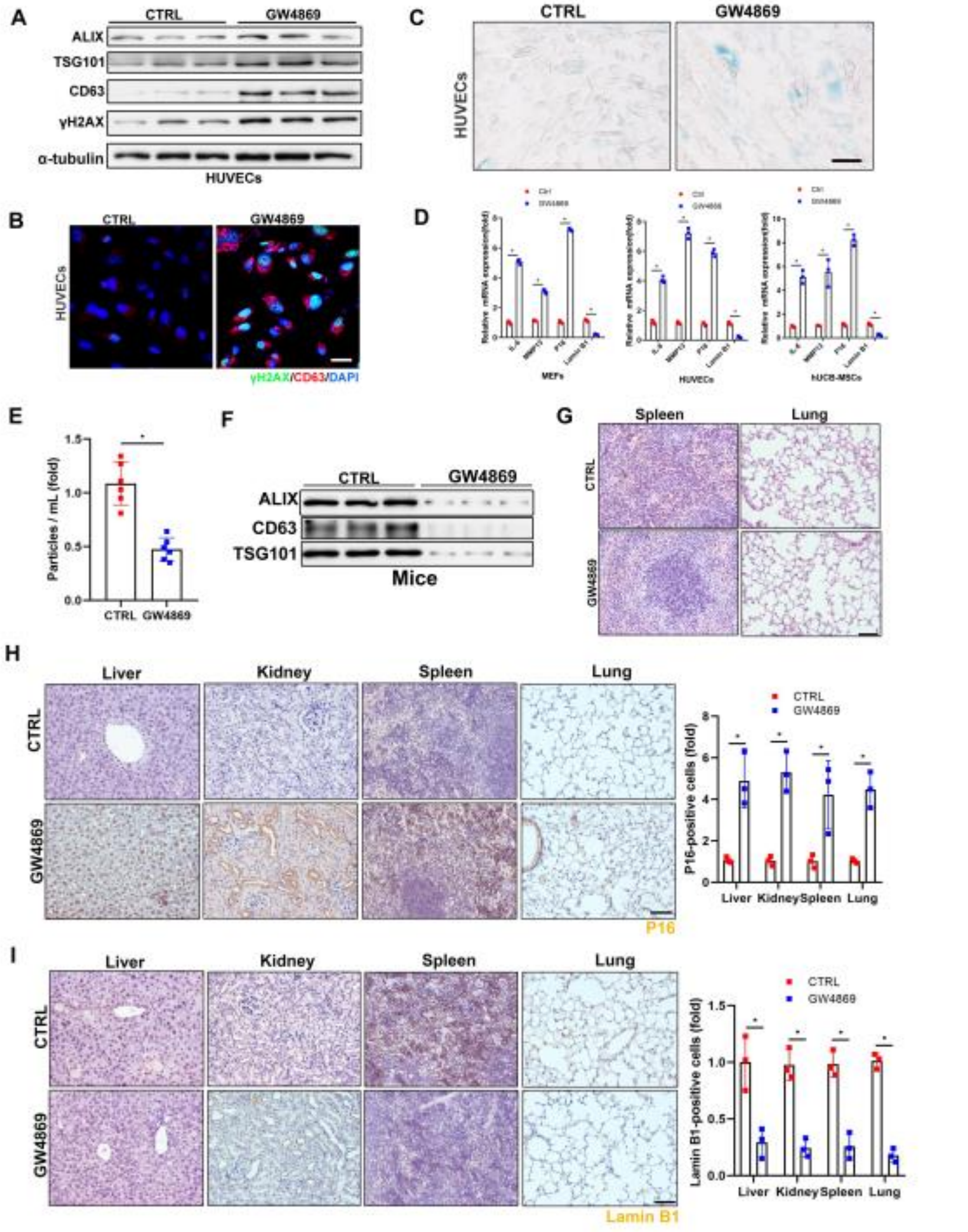

Fig. S1. (A-D) Human umbilical vein endothelial cells (HUVECs) were treated with GW4869 (2 μM) for 5 days. (A) Cell levels of exosome marker proteins and senescence marker proteins were determined by western blot. (B) Cells were fixed and stained with anti-γH2AX (green) and anti-CD63 antibodies (red), and nuclei were stained with DAPI (blue). Confocal microscopic images of stained cells, with quantitative presentation of fold-changes in γH2AX-positive foci in cells. Scale bars = 30 μm. (C) Cells were fixed and stained with

SA-β-gal. Scale bars = 50 μm. (D) Expression levels of *IL6, MMP13, P16*, and *Lamin B1* mRNA were determined by qRT-PCR. *GAPDH* was used as the internal control. Three independent experiments were analyzed. (E-I) 6-month-old mice (n = 6) were treated with GW4869 (2.5 μg/g) or drug vehicle for 2 months *via* the tail intravenous injection. (E) The animals (n = 6) were sacrificed and plasma exosomes were isolated and subjected to NTA. (F) The levels of exosome marker proteins in released exosomes isolated from animal plasma were determined by western blot with quantification. Three independent experiments were analyzed. (G) Representative HE staining images of spleen and lung. Scale bars = 50 μm. (H, I) Tissues were stained with the anti-P16 and anti-lamin B1 antibodies, respectively, for immunohistochemical analysis. Scale bars = 50 μm. Relative positive cells were quantified. Three independent experiments were analyzed. *$p < 0.05$.

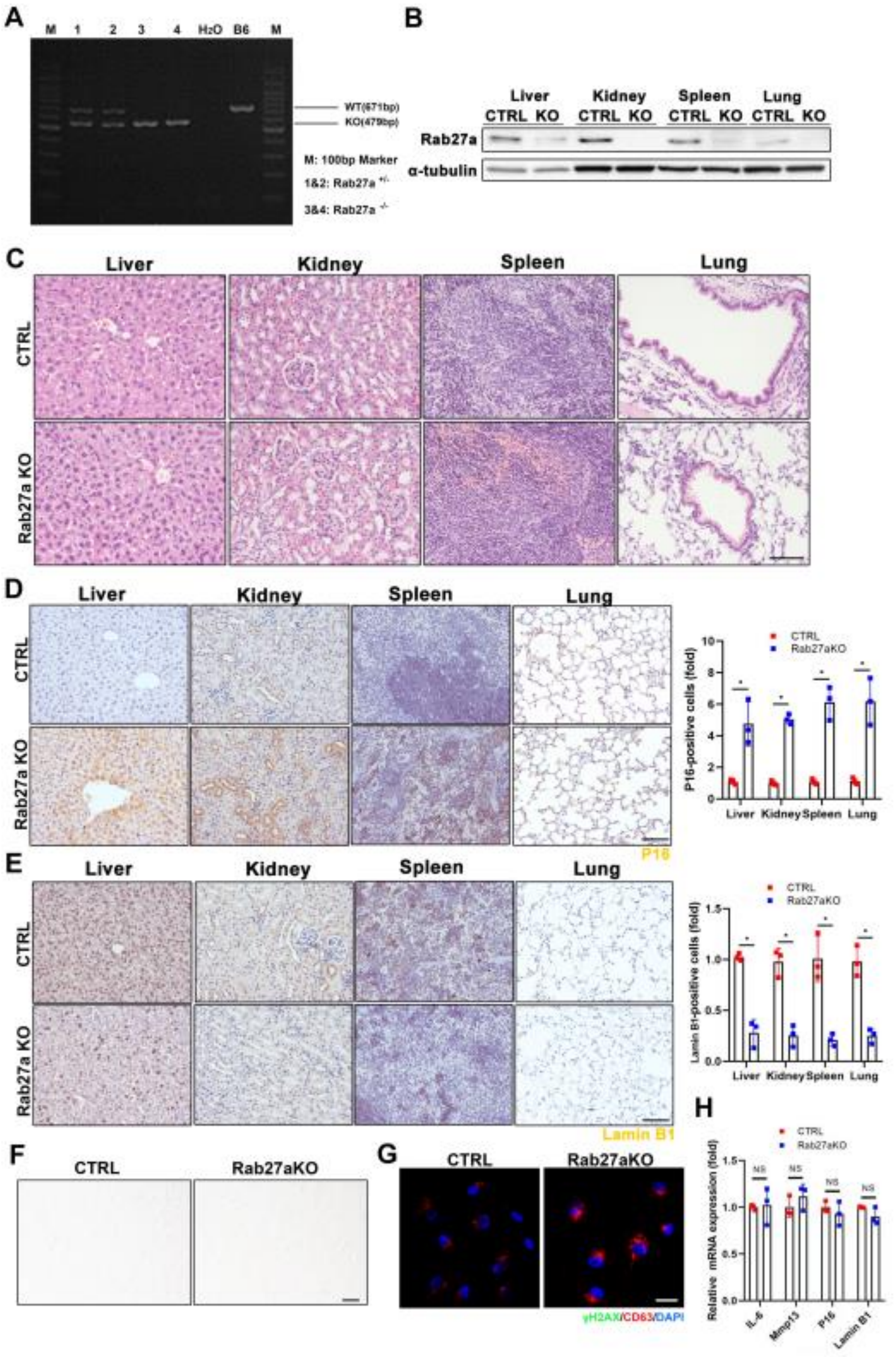

Fig. S2. (A, B) Use the CRISPR/Cas9 system to construct *Rab27a* knockout mice. (A) Genotypes of F2 mice were identified by the agarose gel electrophoresis. (B) Knockout validation was monitored with the Rab27a levels of tissues by western blot. (C-E) *Rab27a* KO mice (6 months old, n = 6) were sacrificed. (C) Representative HE staining images of

tissues. (D, E) Tissues were stained with the anti-P16 and anti-lamin B1 antibodies, respectively, for immunohistochemical analysis. Scale bars = 50 μm. Relative positive cells were quantified. Three independent experiments were analyzed. (F) Young *Rab27a* KO MEFs (P2) were fixed and stained with senescence-associated β-galactosidase (SA-β-gal), scale bars = 50 μm. (G) Confocal microscopic images of stained cells. Young *Rab27a* KO MEFs (P2) were fixed and stained with the anti-γH2AX (green) and anti-CD63 (red), together with DAPI for nuclei (blue), scale bars = 30 μm. (H) The expression of *IL6, MMP13, P16,* and *Lamin B1* mRNA in young *Rab27a* KO MEFs (P2) were determined by qRT-PCR. GAPDH was used as the internal control. Three independent experiments were analyzed. $*p < 0.05$.

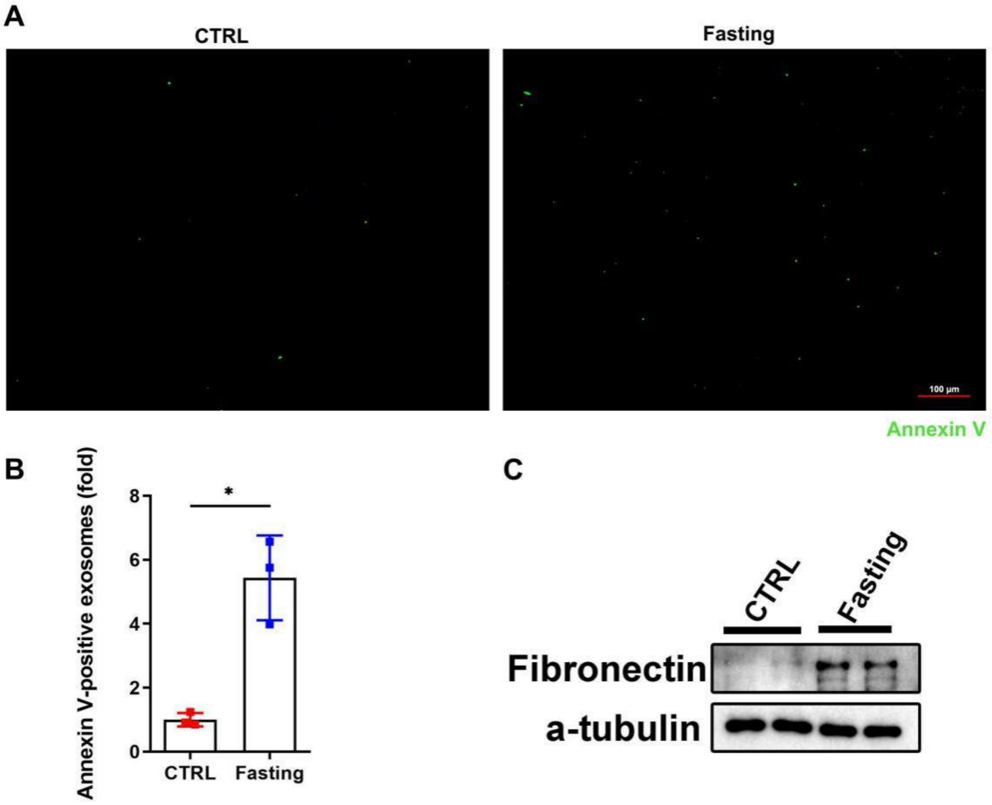

Fig. S3. (A) Levels of phosphatidylserine were imaged and (B) the relative expression from three independent experiments were analyzed. *p < 0.05. (C) Levels of fibronectin were determined by western blot. Three independent experiments were analyzed.

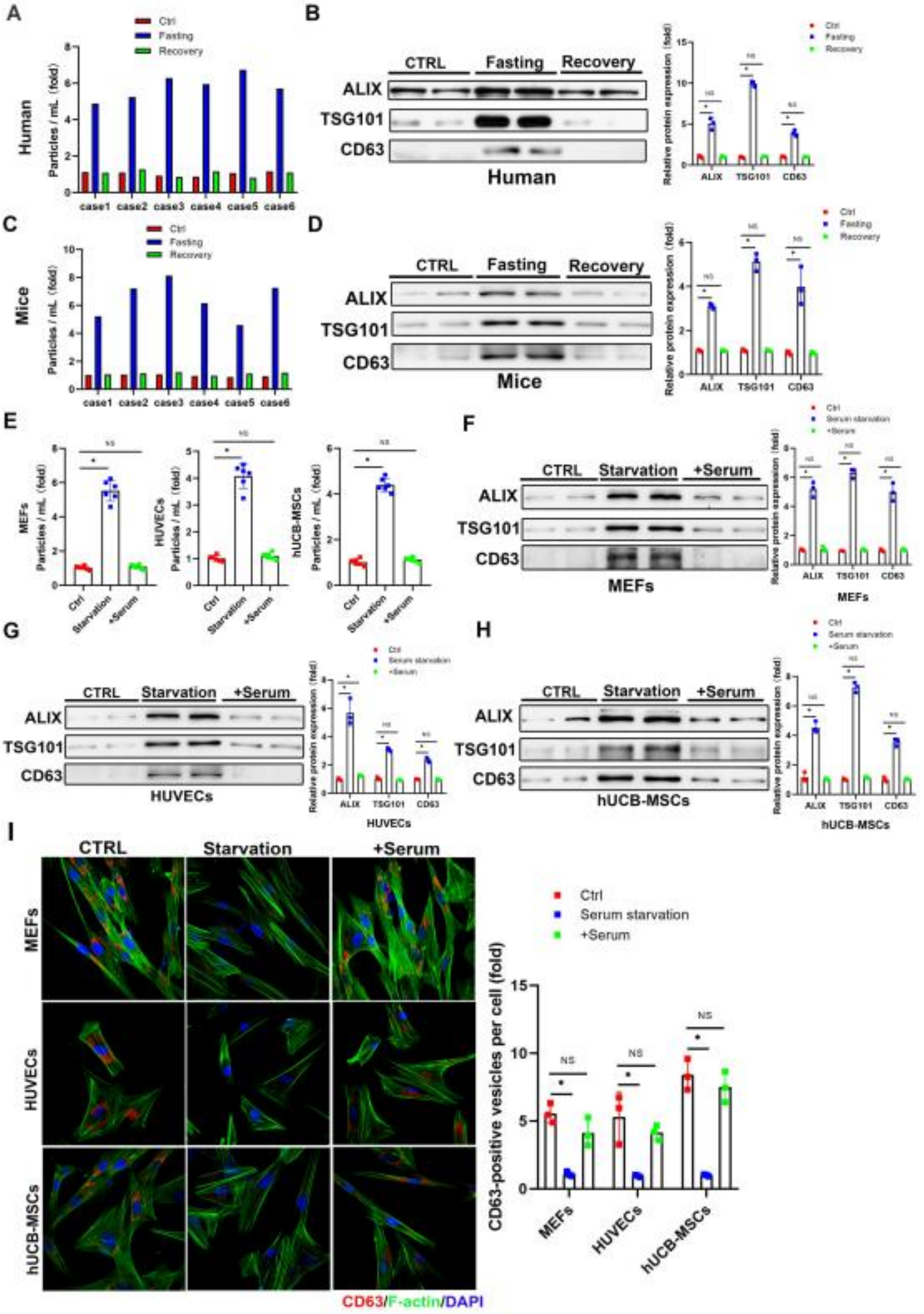

Fig. S4. (A, B) Volunteers (20–30 years old, n = 6) fasted for 24 h and then ate normally. Blood samples were obtained before and after fasting, and 24 h after eating. (A) Plasma exosomes were isolated and subjected to NTA (n = 6). (B) Levels of exosome marker proteins

in plasma exosomes were determined by western blot, with quantification. Three independent experiments were analyzed. (C, D) Six-month-old mice (n = 6) were fasted for 48 h and then fed. Blood samples were obtained before and after fasting, and 24 h after feeding. (C) Plasma exosomes were isolated and subjected to NTA (n = 6). (D) Levels of exosome marker proteins in plasma exosomes were determined by western blot, with quantification. Three independent experiments were analyzed. (E) MEFs, HUVECs, and hUCB-MSCs were cultured in serum-containing media for 24 h followed by serum-deprived media. After incubation for 24 h, the cells were then switched back to serum-containing media for another 24 h. Numbers of exosomes isolated from the culture media of MEFs, HUVECs, and hUCB-MSCs before and after medium switching were determined by NTA (n = 6). $*p < 0.05$. (F–H) Levels of exosome marker proteins in exosomes isolated from the media of MEFs, HUVECs, and hUCB-MSCs were determined by western blot, with quantification. Three independent experiments were analyzed. (I) Confocal microscopic images of stained cells. MEFs, HUVECs, and hUCB-MSCs were fixed and stained with anti-γH2AX (green) and anti-CD63 antibodies (red), and nuclei were stained with DAPI (blue), with quantitative presentation of fold-changes of γH2AX-positive foci in cells. Scale bars = 30 μm. A total of 60 randomly selected cells from three independent experiments were analyzed. $*p < 0.05$.

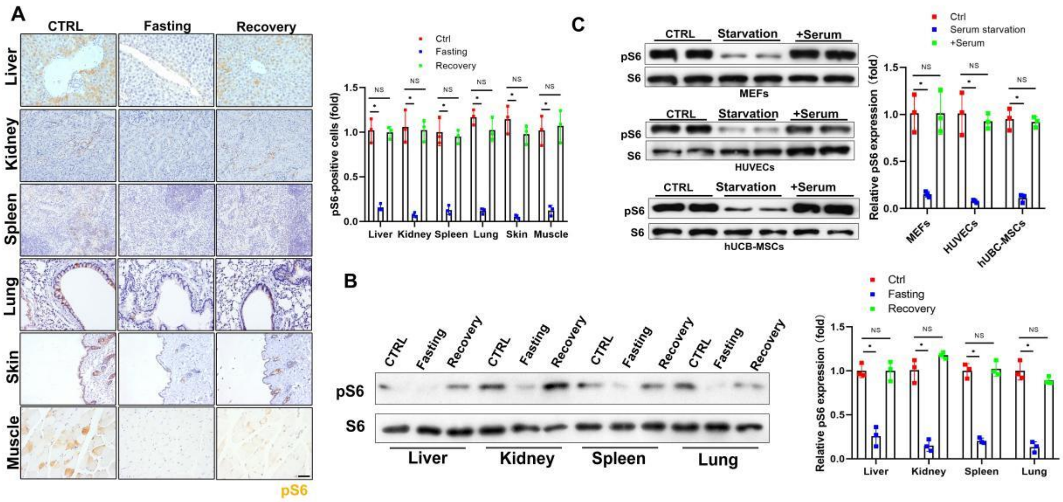

Fig. S5. (A, B) Six-month-old mice (n = 6) were fasted for 2 days and then provided with food. The mice were sacrificed and tissues were collected. (A) Tissues were fixed and stained with the anti-pS6 antibody for immunohistochemical analysis. Three independent experiments were analyzed. (B) mTORC1 activity was monitored by pS6 levels by western blot, with quantification. Three independent experiments were analyzed. (C) MEFs, HUVECs, and hUCB-MSCs were cultured in serum-containing medium for 24 h followed by serum-deprived medium. After incubation for 24 h, the cells were then switched back to serum-containing medium for another 24 h. mTORC1 activity in cell extracts was monitored together with levels of pS6 by western blot, with quantification. Three independent experiments were analyzed. *$p < 0.05$.

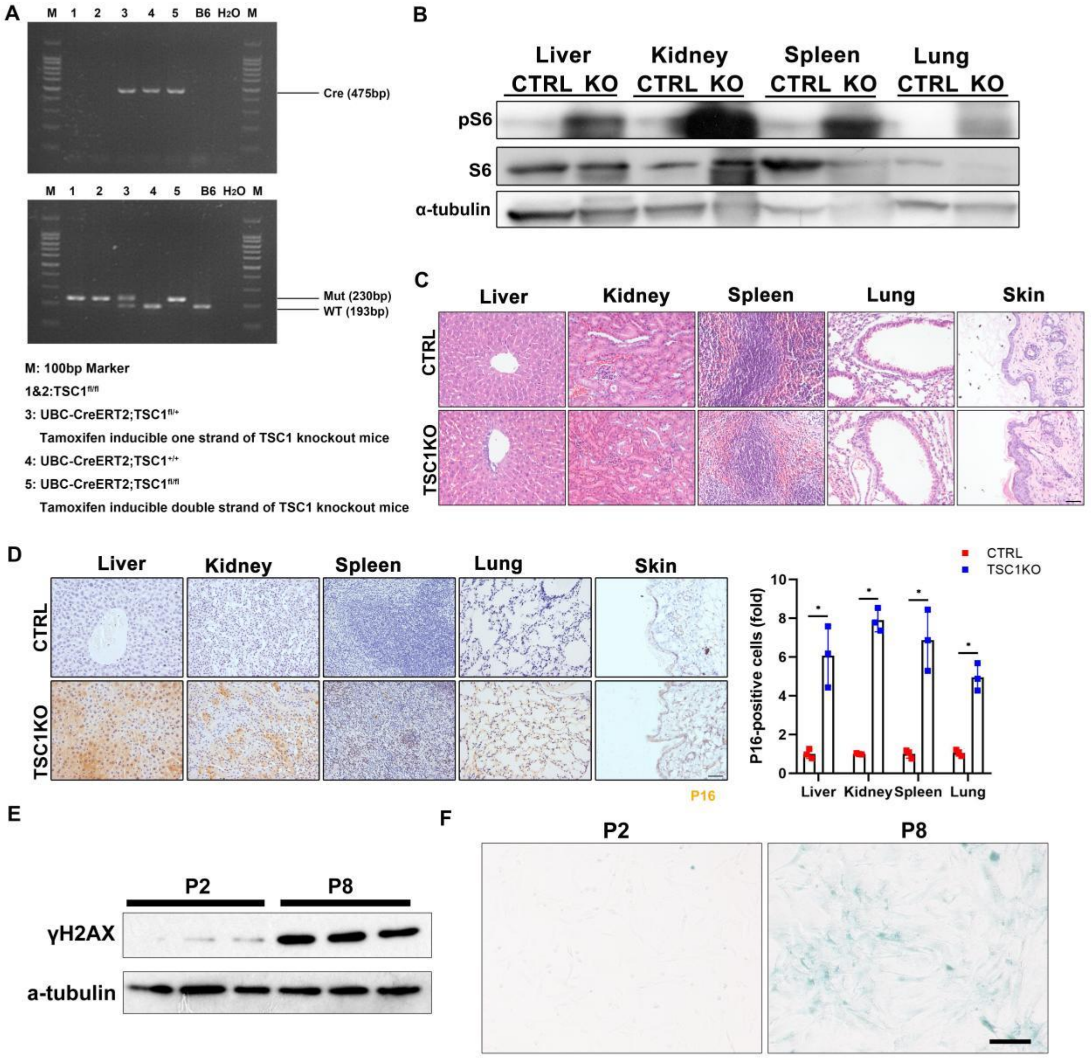

Fig. S6. (A, B) Use the Cre-LoxP recombination system to construct tamoxifen inducible *TSC1* knockout mice. (A) Genotypes of mice were identified by the agarose gel electrophoresis. (B) the mTORC1 activity was monitored with the pS6 levels of tissues by western blot. (C, D) 3-month-old tamoxifen-inducible UBC-Cre-ERT2;*TSC1* fl/fl mice (*TSC1*KO, n = 3) were treated with tamoxifen (100 mg/kg/d) for 5 days. Mice were sacrificed and tissues were collected and fixed. (C) Representative HE staining images of tissues. (D) Tissues were stained with the anti-P16 antibody for immunohistochemical analysis (n = 3), scale bars = 50 μm. Relative positive cells were quantified. *$p < 0.05$. (E) Young (passage 2) and old (passage 8) MEFs levels of senescence marker proteins were determined by western blot. (F) Young (passage 2) and old (passage 8) MEFs were fixed and stained with senescence-associated β-galactosidase (SA-β-gal), scale bars = 50 μm.

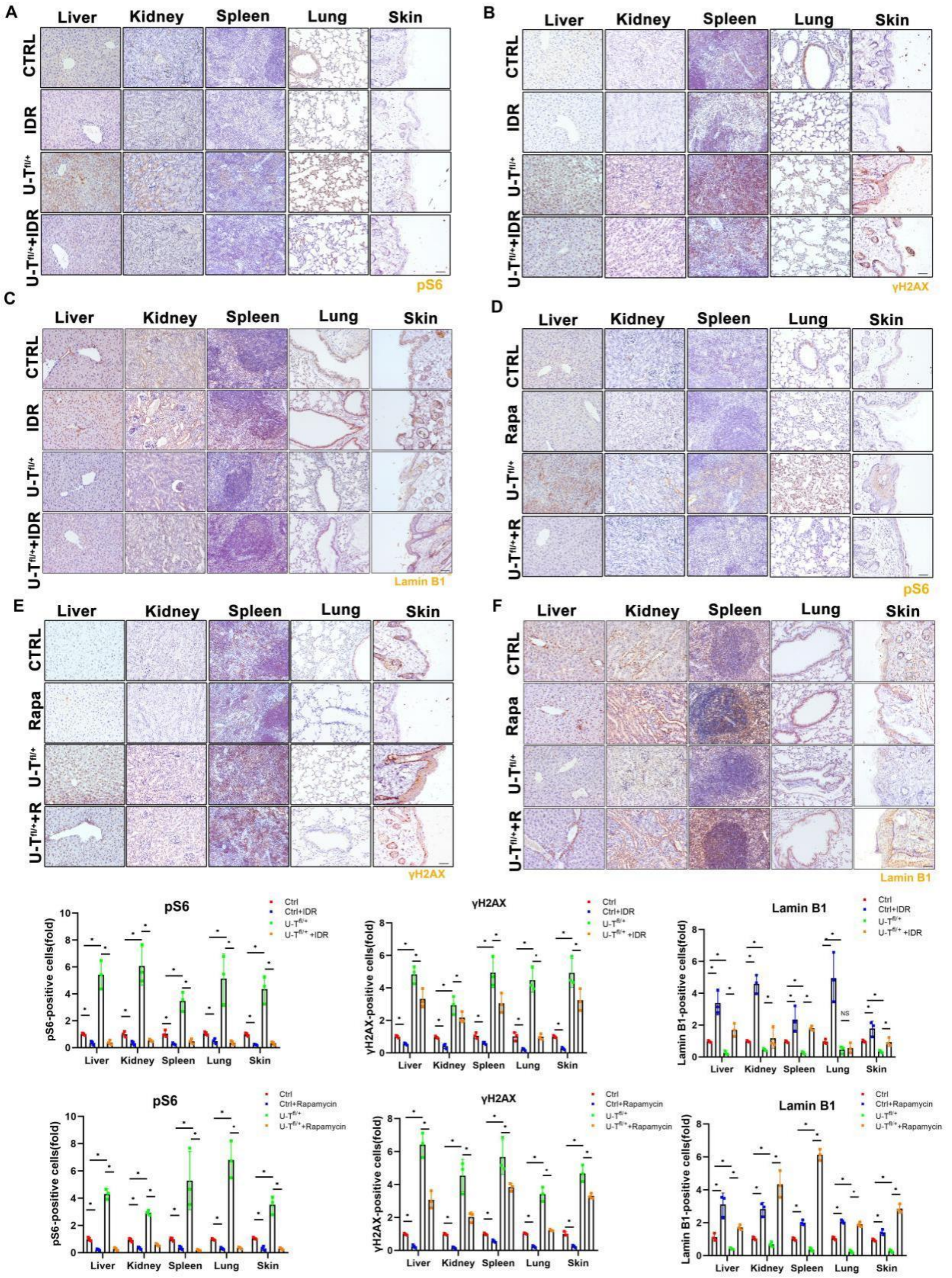

Fig. S7. (A-C) 3-month-old tamoxifen-inducible UBC-Cre-ERT2;*TSC1* fl/+ mice (*U-T* fl/+, n = 3) were under intermittent fasting for 5 months. The animals were sacrificed and tissues were fixed and stained with the anti-pS6, anti-P16 and anti-lamin B1 antibody for immunohistochemical analysis. Three independent experiments were analyzed. (D-F) 3-month-old tamoxifen-inducible UBC-Cre-ERT2; *TSC1* fl/+ mice (*U-T* fl/+, n = 3) were administrated with rapamycin (2.5 mg/kg/d) or drug vehicle for 5 months. The animals (n = 3)

were sacrificed and tissues were fixed and stained with the anti-pS6, anti-γH2AX and anti-lamin B1 antibodies, respectively, for immunohistochemical analysis. Scale bars = 50 μm. Relative positive cells were quantified. *$p < 0.05$.

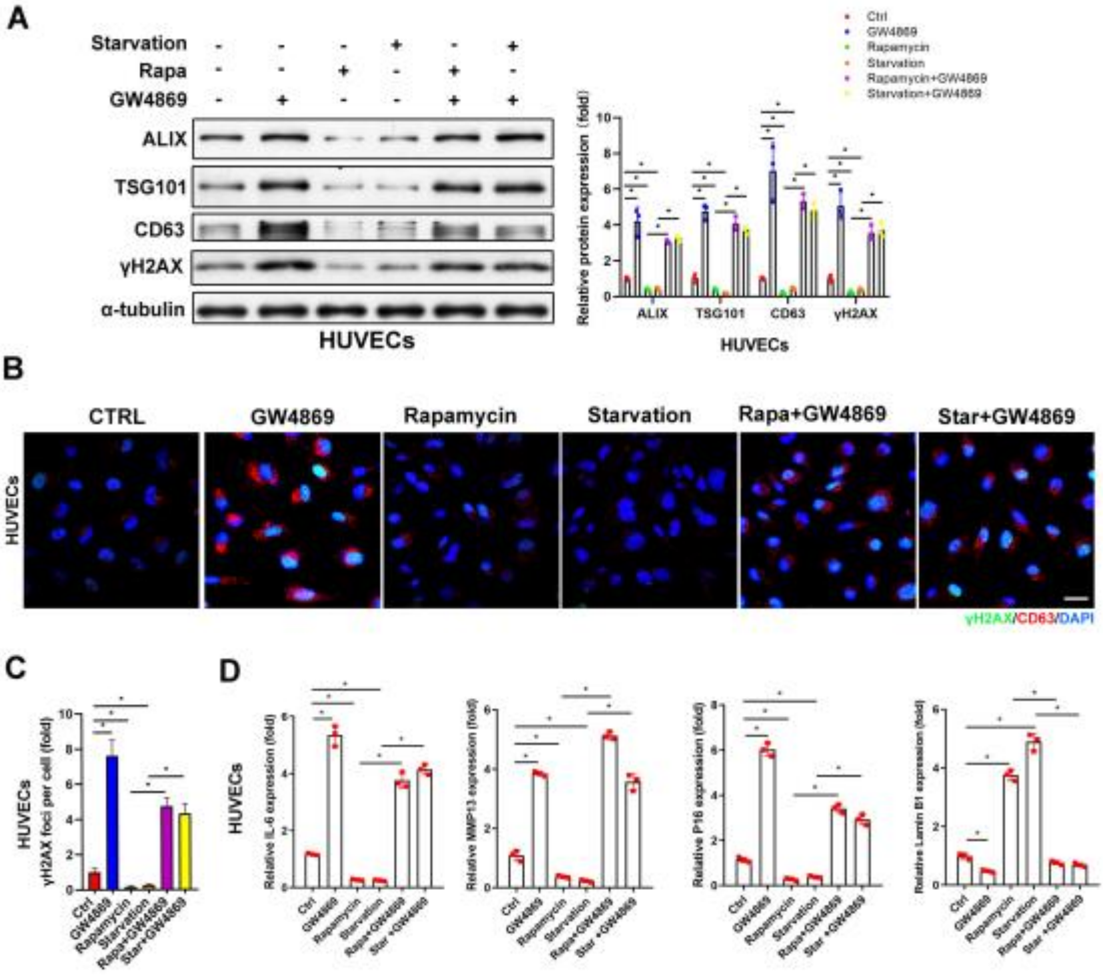

Fig. S8. (A–D) HUVECs were continuously passaged until senescence and then treated with rapamycin (100 nM) or cultured in 1% serum-containing medium for 24 h before treating with GW4869 (2 μM) for 5 days. (A) Levels of exosome marker proteins and senescence marker proteins were determined by western blot with quantification. Three independent experiments were analyzed. (B, C) Confocal microscopic images of stained cells. Cells were fixed and stained with anti-γH2AX (green) and anti-CD63 antibodies (red), and nuclei were stained with DAPI (blue), with quantitative presentation of the fold-changes of γH2AX-positive foci in cells. A total of 60 randomly selected cells from three independent experiments were analyzed. Scale bars = 30 μm. (D) Expression levels of *IL6, MMP13, P16,*

and *Lamin B1* mRNA were determined by qRT-PCR. *GAPDH* was used as the internal control. Three independent experiments were analyzed. *$p < 0.05$.

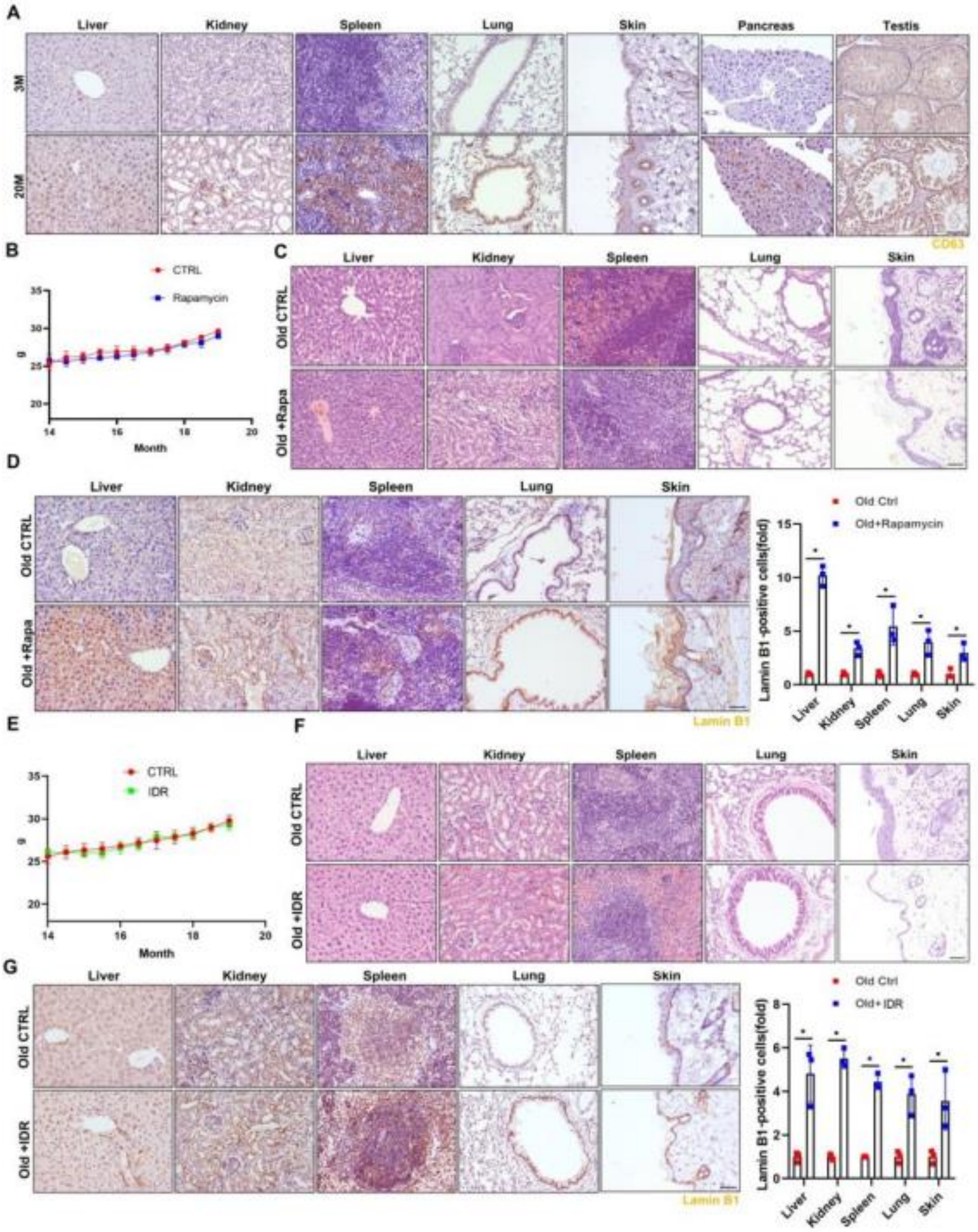

Fig. S9. (A) Representative micrographs of the anti-CD63 antibody immunohistochemistry in tissues of 3-month-old mice (n = 3) and 20-month-old mice (n = 3). (B-D) 14-month-old mice (n = 3) were administrated with rapamycin (2.5 mg/kg/d) or drug vehicle for 5 months. (B) Body weight was monitored every two weeks (n = 3). (C) Representative HE staining images

of tissues. (D) Tissues were stained with the anti-lamin B1 antibody for immunohistochemical analysis (n = 3). (E-G) 14-month-old mice (n = 3) were under intermittent fasting for 5 months. (E) Body weight was monitored every two weeks (n = 3). (F) Representative HE staining images of tissues. (G) Tissues were stained with the anti-lamin B1 antibody for immunohistochemical analysis. Scale bars = 50 μm. Relative positive cells were quantified. Three independent experiments were analyzed. *$p < 0.05$.

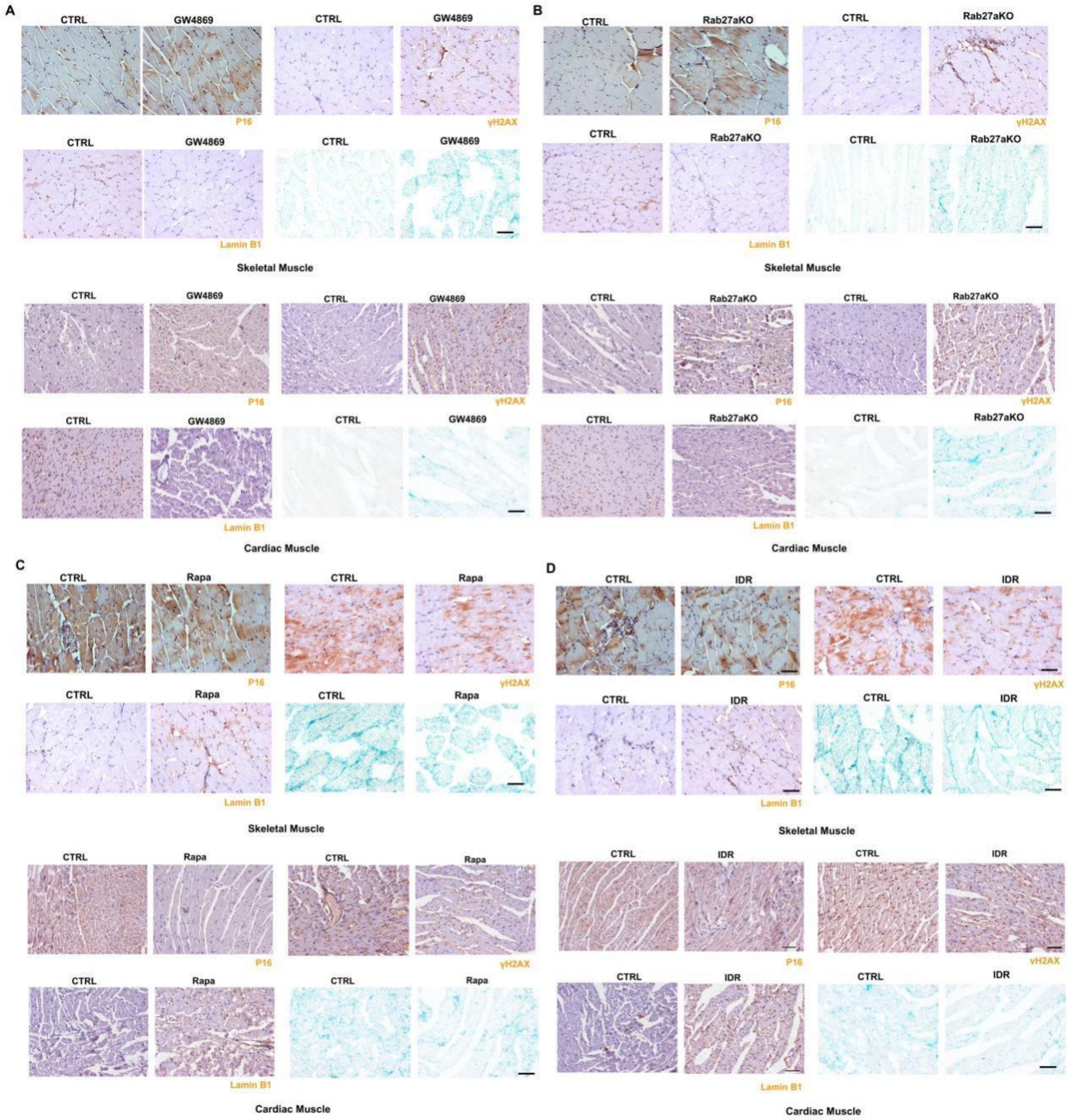

Fig. S10. (A) GW4869-treated mice group, (B) *Rab27a* KO mice group, (C) intermittent dietary restriction group of aged mice, (D) rapamycin-treated group of aged mice (n = 3) were

sacrificed and muscle tissues (skeletal, cardiac) were collected, and stained with senescence-related indicators P16, γH2AX, Lamin B1, and SA-β-gal. Scale bars = 50 μm.

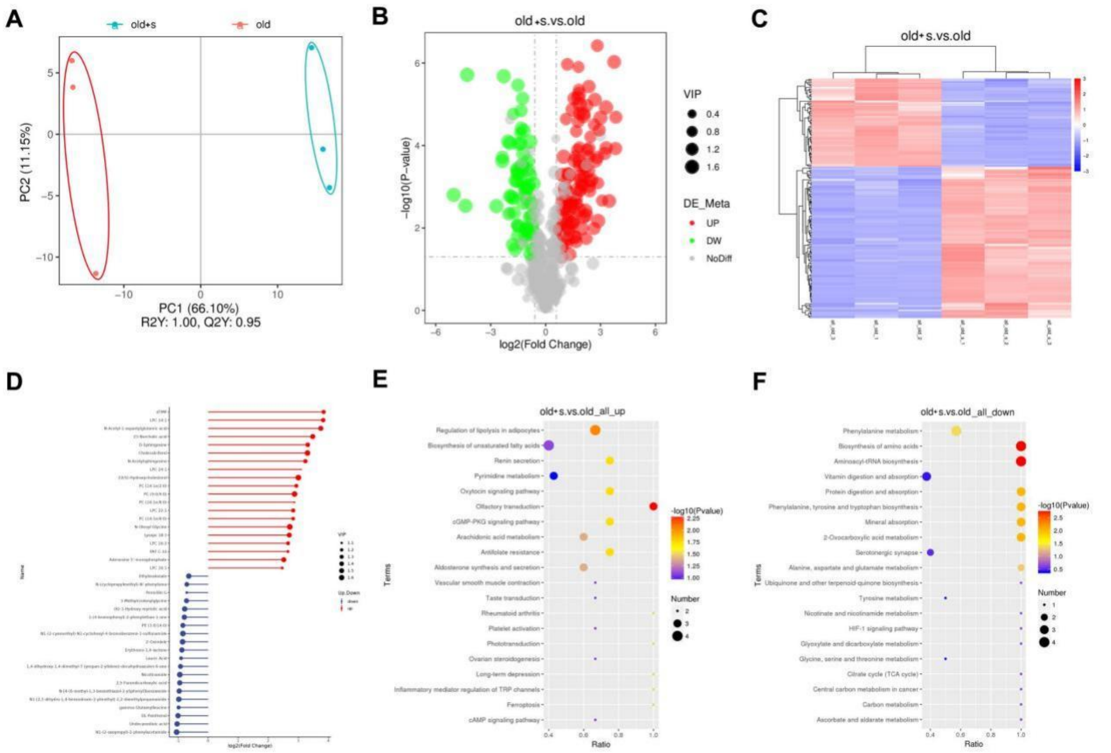

Fig. S11. Old MEFs (passage 8) were cultured in serum-containing media for 24 h. After incubation for 24 h, cells were then switched to serum-deprived media for another 24 h. Exosomes isolated from the culture media of MEFs, before and after medium switching were analyzed and identified by untargeted metabolomics in Novogene (Beijing, China). (A) Partial least-squares discrimination analysis (PLS-DA) of the exosomes isolated from serum-containing media and serum-deprived media (n = 3) of old MEFs (passage 8). Each symbol represents the data of an individual sample. (B) Volcano plots visualize the overall distribution of differential metabolites, with the horizontal coordinate indicating the change in the fold of difference ($\log_2$(Fold Change)) and the vertical coordinate indicating the level of significance of the difference ($-\log_{10}$(P-value)) in different subgroups of metabolites. (C) Heatmap shows differential metabolic production (DMP) in the exosomes isolated from serum-containing media and serum-deprived media of old MEFs (passage 8). (D) Stem plot shows the top 20 differential metabolites in terms of up- and down-regulation. (E) KEGG analysis of these differential (up) metabolic production-enriched biological processes. (F)

KEGG analysis of these differential (down) metabolic production-enriched biological processes.

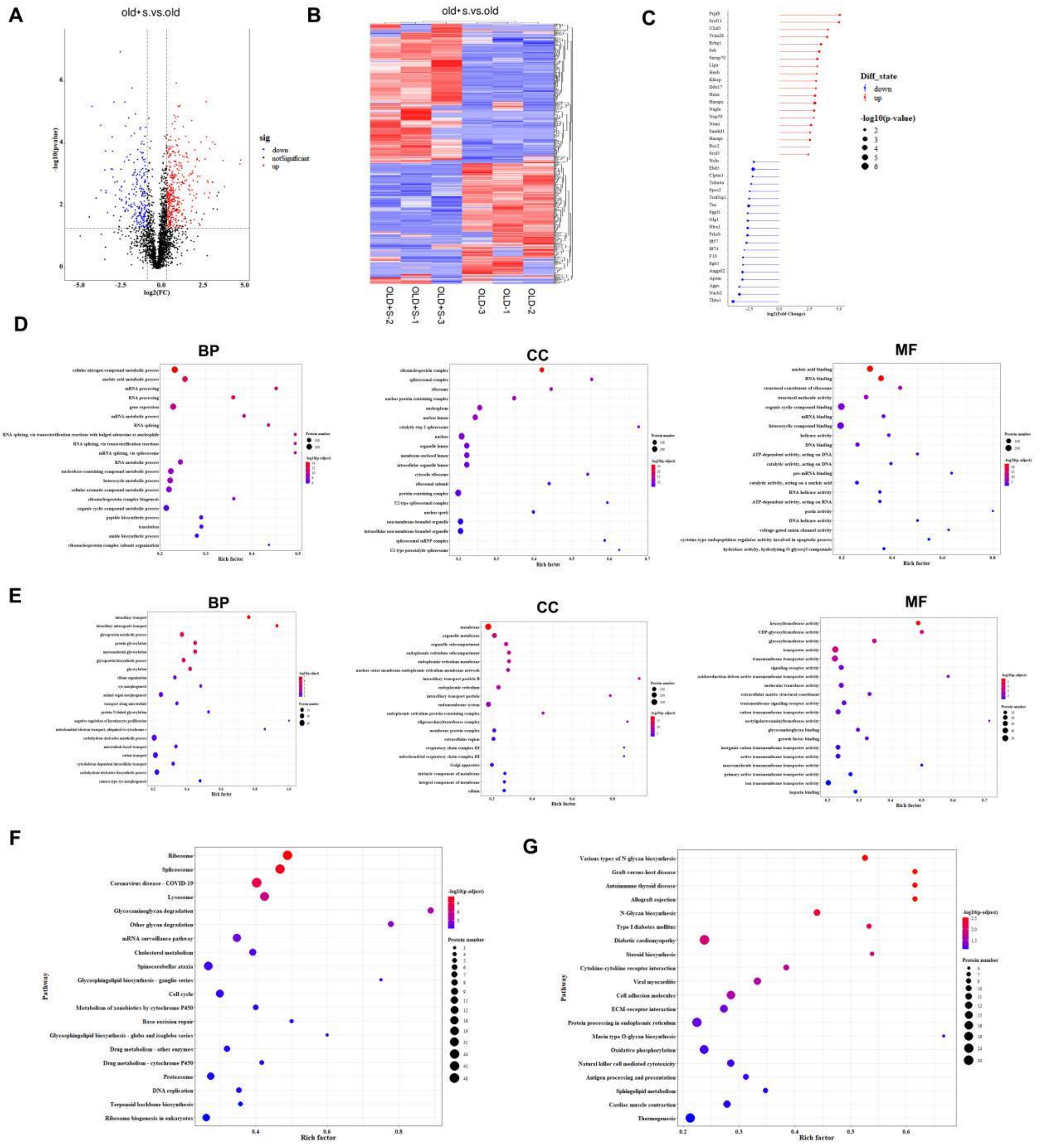

Fig. S12. Old MEFs (passage 8) were cultured in serum-containing media for 24 h. After incubation for 24 h, cells were then switched to serum-deprived media for another 24 h. Exosomes isolated from the culture media of MEFs, before and after medium switching were analyzed and identified by proteomics in Fitgene (Guangzhou, China). (A) Volcano plots visualize the overall distribution of differential proteins, with the horizontal coordinate

indicating the change in the fold of difference (log_2(Fold Change)) and the vertical coordinate indicating the level of significance of the difference ($-log_{10}$(P-value)) in different subgroups of proteins. (B) Heatmap shows differential proteins in the exosomes isolated from serum-containing media and serum-deprived media of old MEFs (passage 8). (C) Stem plot shows the top 20 differential proteins in terms of up- and down-regulation. (D) Bubble diagram of the enrichment distribution of identified proteins (up) in GO functional categories (biological process, cellular component, molecular function). (E) Bubble diagram of the enrichment distribution of identified proteins (down) in GO functional categories (biological process, cellular component, molecular function). (F) KEGG analysis of these differential (up) proteins-enriched biological processes. (G) KEGG analysis of these differential (down) proteins-enriched biological processes.

References

1. Kern S, Eichler H, Stoeve J, Kluter H, & Bieback K (2006) Comparative analysis of mesenchymal stem cells from bone marrow, umbilical cord blood, or adipose tissue. *Stem cells* 24(5):1294-1301.
2. Assouvie A, Daley-Bauer LP, & Rousselet G (2018) Growing Murine Bone Marrow-Derived Macrophages. *Methods in molecular biology* 1784:29-33.
3. Xu J (2005) Preparation, culture, and immortalization of mouse embryonic fibroblasts. *Current protocols in molecular biology* Chapter 28:Unit 28 21.
